# An integrative approach reveals five new species of highland papayas (Caricaceae, *Vasconcellea*) from northern Peru

**DOI:** 10.1371/journal.pone.0242469

**Published:** 2020-12-10

**Authors:** Daniel Tineo, Danilo E. Bustamante, Martha S. Calderon, Jani E. Mendoza, Eyner Huaman, Manuel Oliva

**Affiliations:** Instituto de Investigación para el Desarrollo Sustentable de Ceja de Selva (INDES-CES), Universidad Nacional Toribio Rodríguez de Mendoza, Chachapoyas, Amazonas, Peru; National Agri-Food Biotechnology Institute (NABI) Mohali, INDIA

## Abstract

The assignment of accurate species names is crucial, especially for those with confirmed agronomic potential such as highland papayas. The use of additional methodologies and data sets is recommended to establish well-supported boundaries among species of *Vasconcellea*. Accordingly, six chloroplast (*trnL*-*trnF*, *rpl20*-*rps12*, *psbA*-*trnH* intergenic spacers, *matK* and *rbcL* genes) and nuclear (ITS) markers were used to delimit species in the genus *Vasconcellea* using phylogeny and four DNA-based methods. Our results demonstrated congruence among different methodologies applied in this integrative study (i.e., morphology, multilocus phylogeny, genetic distance, coalescence methods). Genetic distance (ABGD, SPN), a coalescence method (BPP), and the multilocus phylogeny supported 22*–*25 different species of *Vasconcellea*, including the following five new species from northern Peru: *V*. *badilloi* sp. nov., *V*. *carvalhoae* sp. nov., *V*. *chachapoyensis* sp. nov., *V*. *pentalobis* sp. nov., and *V*. *peruviensis* sp. nov. Genetic markers that gave better resolution for distinguishing species were ITS and *trn*L-*trn*F. Phylogenetic diversity and DNA-species delimitation methods could be used to discover taxa within traditionally defined species.

## Introduction

The family Caricaceae is composed of six genera containing 35 species that are distributed from southern Mexico to northern Chile [1, 2]. Two of these genera, namely *Carica* L. and *Horovitzia* Badillo are monospecific. The former is considered the most economically important and is distributed in tropical and subtropical America [3], whereas the latter is endemic to Mexico [4]. Three additional genera are *Cylicomorpha* Urban, *Jacaratia* A. DC, and *Jarilla* Rusby. The first one has two species and is the only genus restricted to the African premontane wet forestst [2]. The second one comprises seven species distributed along South America [2]. The latter one encompasses three species distributed along Pacific Coast from northern Mexico to El Salvador [5]. The remaining genus, namely *Vasconcellea* Saint-Hilaire, is the largest one in this family encompassing 20 species and 1 hibrid (*V*. x *heibornii*) distributed mainly from Ecuador to Peru [3, 6].

Initially, *Vasconcellea* was embedded into the genus *Carica* [7]; however, molecular analyses confirmed that these genera were not monophyletic [8, 9], and *Vasconcellea* was restored as a different genus [6]. The genus *Vasconcellea* is characterized by simple, lobed, or palm-lobed leaves with five to six main veins [10]. Besides, flowers have a corolla with curved lobules to the left, linear stigmas, five locule ovaries, and scattered ovules in two juxtaposed divisions [10]. The species richness of *Vasconcellea* shows that northern Andes are the areas with the highest diversity [1, 10]. *Vasconcellea* species are distributed from the dry slopes of the Andes in Ecuador, Colombia and Peru (3,500 m.a.s.l.) to the lowlands of Panama to southern Brazil and called highland papayas due to their climatic preferences [1]. Highland papayas have a number of desirable characteristics, such as disease resistance, cold tolerance, high latex enzymatic activity, and high protein and vitamin contents [10], which suggest their agronomic potential, especially in Andean towns [11]. Currently, eigth species of the genus *Vasconcellea* have been reported from northern (Amazonas, Cajamarca) to southern Peru (Moquegua): *V*. *candicans* (A.Gray) A. DC, *V*. *glandulosa* A. DC, *V*. *microcarpa* (Jacq.) A. DC, *V*. *monoica* (Desf.) A. DC, *V*. *parviflora* A. DC, *V*. *pubescens* A. DC, *V*. *quercifolia* A. St.-Hil., and *V*. *weberbaueri* (Harms) V.M. Badillo [2, 12–14].

The evolutionary history of these taxa might be misunderstood by recognizing distinct clades in single gene trees as species [15]. Therefore, the use of multilocus sequence data is crucial in the establishment of robust species boundaries [16, 17]. Several molecular-phylogenetic analyses of Caricaceae have been undertaken using isozymes, RFLP, and AFLP but none included representatives of all genera [8, 9, 14, 18–20]. Nuclear and plastid DNA sequences from all of the family’s extant species was compiled [2] and the evolutionary relationships within the family Caricaceae have therefore clarified. These chloroplast (*trnL-trnF*, *rpl20-rps12*, *psbA-trnH* intergenic spacers, *matK* and *rbcL* genes) and nuclear sequences (ITS) have been recommended to assess inter- and intraspecific relationships among species of Caricaceae [2, 9]. Additionally, estimating species trees and establishing species boundaries among different taxa are challenging [15, 17]. This has been overcome by methods that encompass genetic distance and coalescent approaches, which have proven very useful and been widely used for a range of taxa [15, 17–20]. Accordingly, the use of several methodologies and data sets to delimit species (i.e., integrative approaches) is highly recommended, and subsequently, the achievement of congruent results across the methods is likely to prove most useful for framing reliably supported species boundaries [21–25].

In this study, we analysed species of the genus *Vasconcellea*, including new unreported taxa from northern Peru, based on an integrative approach (i.e., morphological observations, phylogenetic inferences, and DNA-species delimitation methods). Six molecular markers (ITS, *matK*, *psbA-trnH*, *rbcL*, *rpl20-rps12*, *trnL-trnF*) were used to examine the phylogenetic relationships and assess boundaries of species within the genus *Vasconcellea*.

## Materials and methods

### Collection of specimens

**Fig 1:**
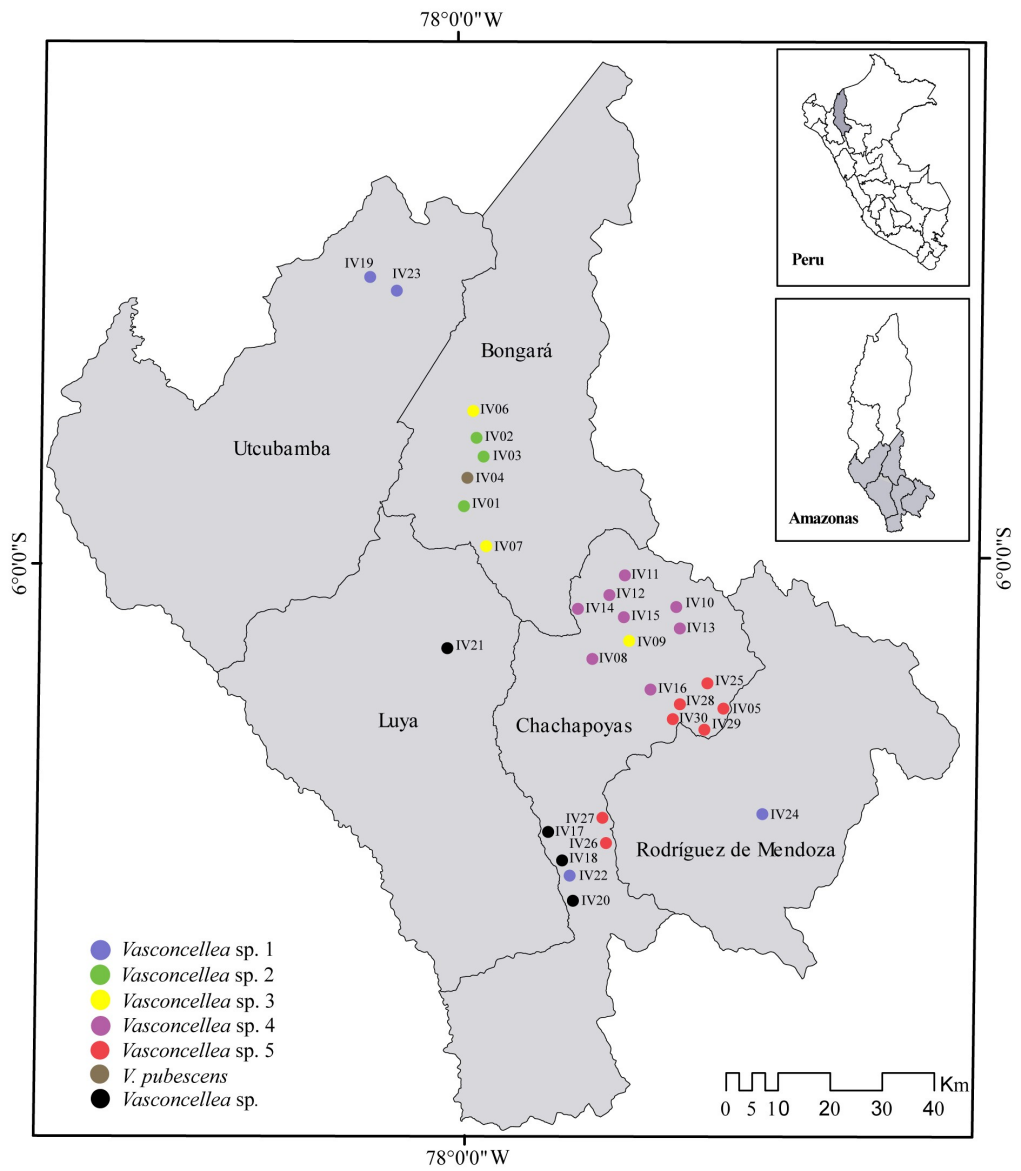
Collections of the 30 specimens of the genus *Vasconcellea* from the Region Amazonas, northern Peru. The national, provincial and district boundaries were obtained from the Geoportal of the National Geographic Institute of Peru (IGN) in shapefile format with a DATUM WGS 1984 for illustrative purposes only.

A total of 30 specimens of highland papayas were sampled from five provinces along the Region Amazonas, in northern Peru (Bongará, Chachapoyas, Luya, Rodriguez de Mendoza, Utcubamba; Fig 1). Permit of scientific research of wild flora (D000134-MINAGRI-SERFOR-DGGSPFFS-DGSPF) was provided by Servicio Nacional Forestal y de Fauna Silvestre (SERFOR). Tissue samples of approximately 50 mm^2^ were taken from leaf tips for genetic analyses and placed in prelabelled 1.5 mL Safelock Eppendorf tubes. For each site, the date, time, and GPS coordinates were recorded. Photographs were taken to record sampling locations and site features. Samples were morphologically characterized according to Badillo [4, 6] and deposited in the herbarium of Universidad Nacional Toribio Rodríguez de Mendoza (CHAX), Peru (Table 1) [26]. Furthermore, records and morphology of *Vasconcellea* species were revised from databases and collections as JSTOR Global Plants (https://plants.jstor.org/), the New York Botanical Garden Steere herbarium (http://sweetgum.nybg.org/science/), the Global Biodiversity Information Facility (https://www.gbif.org/), and Tropicos from Missouri Botanical Garden (http://www.tropicos.org).

**Table 1:** List of samples of highland papayas collected in Region Amazonas, northern Peru.

Species	Code	Herbario Voucher	Place	Date	Elevation (m.a.s.l)	Latitude (South)	Longitude (West)
*V*. *badilloi*	IV06	CHAX224	Pomacochas, Bongará	13 Sep. 2018	2280	5°48'53''	77°57'24"
*V*. *badilloi*	IV07	CHAX225	Cuchulia, Bongará	13 Sep. 2018	1386	5°59'44''	77°58'30''
*V*. *badilloi*	IV09	CHAX226	Quinjalca, Chachapoyas	20 Sep. 2018	3143	6°05'33"	77°40'39"
*V*. *carvalhoae*	IV01	CHAX227	Pomacochas, Bongará	13 Sep. 2018	2401	5°49'45"	77°58'12"
*V*. *carvalhoae*	IV02	CHAX228	Pomacochas, Bongará	13 Sep. 2018	2263	5°49'08.7"	77°57'39.3"
*V*. *carvalhoae*	IV03	CHAX229	Pomacochas, Bongará	13 Sep. 2018	2236	5°49'37''	77°58'01''
*V*. *chachapoyensis*	IV08	CHAX230	Quinjalca, Chachapoyas	20 Sep. 2018	3130	6°05'30.4"	77°40'30.4"
*V*. *chachapoyensis*	IV10	CHAX231	Granada, Chachapoyas	20 Sep. 2018	2996	6°06'12''	77°37'47''
*V*. *chachapoyensis*	IV11	CHAX232	Olleros, Chachapoyas	20 Sep. 2018	3041	6°03'07''	77°38'54''
*V*. *chachapoyensis*	IV12	CHAX233	Olleros, Chachapoyas	20 Sep. 2018	3031	6°03'13.2"	77°38'47.3"
*V*. *chachapoyensis*	IV13	CHAX234	Granada, Chachapoyas	20 Sep. 2018	3017	6°06'10''	77°37'39''
*V*. *chachapoyensis*	IV14	CHAX235	Asunción, Chachapoyas	20 Sep. 2018	2821	6°01'56.6"	77°42'37.1"
*V*. *chachapoyensis*	IV15	CHAX236	Quinjalca, Chachapoyas	20 Sep. 2018	3150	6°05'25''	77°40'46''
*V*. *chachapoyensis*	IV16	CHAX237	San José, Chachapoyas	20 Sep. 2018	2200	6°16'59.2''	77°33'31.7"
*V*. *pentalobis*	IV05	CHAX238	Ocol, Chachapoyas	05 Sep. 2018	2297	6°14'49''	77°32'50''
*V*. *pentalobis*	IV25	CHAX239	Ocol, Chachapoyas	08 Feb. 2019	2406	6°15'35''	77°32'45''
*V*. *pentalobis*	IV26	CHAX240	Cuchapata, Chachapoyas	08 Feb. 2019	2523	6°28'25''	77°42'13''
*V*. *pentalobis*	IV27	CHAX241	Cuchapata, Chachapoyas	15 Feb. 2019	2342	6°28'25''	77°41'51''
*V*. *pentalobis*	IV28	CHAX242	Izcuchaca, Chachapoyas	15 Feb. 2019	2385	6°20'08''	77°31'49''
*V*. *pentalobis*	IV29	CHAX243	Izcuchaca, Chachapoyas	15 Feb. 2019	2321	6°20'41''	77°31'17''
*V*. *pentalobis*	IV30	CHAX244	Izcuchaca, Chachapoyas	15 Feb. 2019	2659	6°18'59''	77°33'21''
*V*. *peruviensis*	IV19	CHAX245	Buenos Aires, Utcubamba	12 Aug. 2019	1571	5°40'33.1"	78°20'23.8"
*V*. *peruviensis*	IV22	CHAX246	Cueyqueta, Chachapoyas	16 Oct. 2018	2557	6°31'33.0"	77°48'50.2"
*V*. *peruviensis*	IV23	CHAX247	Buenos Aires, Utcubamba	12 Aug. 2018	1538	5°40'04''	78°20'17''
*V*. *peruviensis*	IV24	CHAX248	Santa Rosa, Rodríguez de Mendoza	16 Aug. 2018	1887	6°26'35.4"	77°28'44.9"
*V*. *pubescens*	IV04	CHAX249	Pomacochas, Bongará	12 Sep. 2019	2285	5°48'31"	77°57'09"
*V*. *stipulata*	IV17	CHAX250	Péngote, Chachapoyas	26 Oct. 2018	2523	6°32'58.1"	77°48'52.4"
*V*. *stipulata*	IV18	CHAX251	Péngote, Chachapoyas	26 Oct. 2018	2422	6°32'23''	77°48'56''
*V*. *stipulata*	IV20	CHAX252	Cueyqueta, Chachapoyas	26 Oct. 2018	2557	6°31'33.0"	77°48'50.2"
*V*. *stipulata*	IV21	CHAX253	Lamud, Luya	30 Oct. 2018	2311	6°08'22.9"	77°57'03.1"

### DNA sequencing and alignment preparation

Genomic DNA was extracted from leaf tissue using the NucleoSpin Plant II Kit (Macherey-Nagel, Düren, Germany) following the manufacturer’s instructions. Six molecular markers were sequenced (ITS, *matK*, *psbA-trnH*, *rbcL*, *rpl20-rps12*, *trnL-trnF*). Each gene was amplified using polymerase chain reaction (PCR) with MasterMix (Promega, Wisconsin, USA) in the following reaction mixture: 10 ng of DNA and 0.25–0.5 pmol of forward and reverse primers for a total volume of 10 μl. The PCR protocols followed Bustamante et al. [17, 27], and primer combinations are summarized in S1 Table. The sequences of the forward and reverse strands were determined commercially by Macrogen Inc. (Macrogen, Seoul, Korea). New generated sequences from the six markers were deposited in GenBank. These sequences and others obtained from GenBank (Table 2) were initially aligned with Muscle algorithms [28] and were adjusted manually with MEGA6 software [29].

**Table 2:** List of species used in the molecular analyses.

Specie	Country	Voucher	ITS	*mat*K	*psb*A-*trn*H	*rbc*L	*rpl*20*-rps*12	*trn*L*-trn*F
*Carica papaya*	Guatemala	KJ399	AY461564	JX092002	JX091963	JX091913	JX091875	JX091823
	Ecuador	RPEH57	JX092051	JX092003	AY847053	JX091914	JX091874	DQ061124
*Cylicomorpha parviflora*	Tanzania	MMA3212	JX092052	JX092004	JX091964	JX091915	JX091876	JX091824
*Cylicomorpha solmsii*	Cameroon	GJP2115	JX092053	JX092005	JX091965	JX091916	JX091877	JX091825
*Horovitzia cnidoscoloides*	Mexico	TRC8167	JX092054	JX092006	JX091966	JX091917	JX091878	JX091826
*Jacaratia corumbensis*	Paraguay	FK1468	JX092056	JX092008	JX091969	JX091918	JX091879	JX091829
*Jacaratia dolichaula*	Mexico	CJI4785	JX092058	JX092010	JX091970	JX091920	JX091881	JX091832
*Jacaratia spinosa*	Peru	HE1348	JX092062	JX092015	JX091972	JX091925	JX091883	JX091836
*Jacaratia* sp.	Peru	HE1365	JX092063	JX092013	JX091974	JX091923	JX091882	JX091827
*Jarilla caudata*	Mexico	LJA20002	JX092065	JX092016	JX091975	JX091926	JX091885	JX091839
*Jarilla chocola*	Mexico	LEJ31	JX092064	JX092017	JX091977	JX091927	JX091884	JX091838
*Jarilla heterophylla*	Mexico	LJA20002	JX092066	JX092018	JX091978	JX091928	JX091886	JX091840
***V*. *badilloi***	Peru	IV06	x	**MT823587**	**MT823611**	**MT823641**	**MT823671**	**MT823701**
	Peru	IV07	x	**MT823588**	**MT823612**	**MT823642**	**MT823672**	**MT823702**
	Peru	IV09	x	**MT823590**	**MT823614**	**MT823644**	**MT823674**	**MT823704**
*V*. *candicans*	Peru	SL1201	JX092074	JX092025	JX091986	JX091936	JX091892	JX091848
***V*. *carvalhoae***	Peru	IV01	x	**MT823582**	**MT823606**	**MT823636**	**MT823666**	**MT823696**
	Peru	IV02	**MT808984**	**MT823583**	**MT823607**	**MT823637**	**MT823667**	**MT823697**
	Peru	IV03	x	**MT823584**	**MT823608**	**MT823638**	**MT823668**	**MT823698**
*V*. *cauliflora*	Guatemala	SPC89272	JX092075	JX092028	JX091987	JX091939	JX091894	JX091850
***V*. *chachapoyensis***	Peru	IV08	**MT808987**	**MT823589**	**MT823613**	**MT823643**	**MT823673**	**MT823703**
	Peru	IV10	**MT808988**	**MT823591**	**MT823615**	**MT823645**	**MT823675**	**MT823705**
	Peru	IV11	**MT808989**	**MT823592**	**MT823616**	**MT823646**	**MT823676**	**MT823706**
	Peru	IV12	**MT808990**	**MT823593**	**MT823617**	**MT823647**	**MT823677**	**MT823707**
	Peru	IV13	**MT808991**	**MT823594**	**MT823618**	**MT823648**	**MT823678**	**MT823708**
	Peru	IV14	x	**MT823595**	**MT823619**	**MT823649**	**MT823679**	**MT823709**
	Peru	IV15	**MT808992**	**MT823596**	**MT823620**	**MT823650**	**MT823680**	**MT823710**
	Peru	IV16	**MT808993**	**MT823597**	**MT823621**	**MT823651**	**MT823681**	**MT823711**
*V*. *chilensis*	Chile	FCsn	JX092076	JX092030	JX091990	JX091941	JX091895	JX091852
*V*. *crassipetala*	Ecuador	RPEH282	AY461530	AY461559	AY847039	JX091942	JX091896	DQ061132
*V*. *glandulosa*	Argentina	NLJ8655	JX092077	JX092033	JX091991	JX091943	JX091897	JX091854
*V*. *goudotiana*	Colombia	RPEH285	AY461540	JX092035	AY847035	JX091945	JX091899	DQ061135
*V*. *x heilbornii*	Ecuador	RPEH155	AY461528	JX092037	x	JX091947	x	DQ061127
*V*. *horovitziana*	Ecuador	RM262683	AY461543	AY461566	AY847036	x	x	DQ061141
*V*. *longiflora*	Ecuador	RPEH228	AY461542	AY461557	AY847037	x	x	DQ061131
*V*. *microcarpa*	Ecuador	RPEH225	AY461536	AY461563	AY847052	JX091948	x	DQ061130
*V*. *monoica*	Ecuador	RPEH58	AY461537	JX092039	AY847032	JX091950	JX091901	DQ061119
*V*. *omnilingua*	Ecuador	RPEH238	AY461534	JX092040	AY847042	JX091951	JX091902	DQ061120
*V*. *palandensis*	Ecuador	RPEH66	AY461535	JX092041	AY847047	JX091952	JX091903	DQ061140
*V*. *parviflora*	Ecuador	RPEH45	AY461526	JX092043	AY847048	JX091954	JX091905	DQ061122
***V*. *pentalobis***	Peru	IV05	**MT808986**	**MT823586**	**MT823610**	**MT823640**	**MT823670**	**MT823700**
	Peru	IV25	**MT808999**	x	**MT823630**	**MT823660**	**MT823690**	**MT823720**
	Peru	IV26	**MT809000**	x	**MT823631**	**MT823661**	**MT823691**	**MT823721**
	Peru	IV27	**MT809001**	x	**MT823632**	**MT823662**	**MT823692**	**MT823722**
	Peru	IV28	**MT809002**	x	**MT823633**	**MT823663**	**MT823693**	**MT823723**
	Peru	IV29	**MT809003**	x	**MT823634**	**MT823664**	**MT823694**	**MT823724**
	Peru	IV30	**MT809004**	x	**MT823635**	**MT823665**	**MT823695**	**MT823725**
***V*. *peruviensis***	Peru	IV19	**MT808994**	**MT823600**	**MT823624**	**MT823654**	**MT823684**	**MT823714**
	Peru	IV22	**MT808996**	**MT823603**	**MT823627**	**MT823657**	**MT823687**	**MT823717**
	Peru	IV23	**MT808997**	**MT823604**	**MT823628**	**MT823658**	**MT823688**	**MT823718**
	Peru	IV24	**MT808998**	**MT823605**	**MT823629**	**MT823659**	**MT823689**	**MT823719**
*V*. *pubescens*	Peru	FHsn	JX092082	JX092044	KU664502	JX091955	JX091906	JX091865
	Peru	IV04	**MT808985**	**MT823585**	**MT823609**	**MT823639**	**MT823669**	**MT823699**
*V*. *pulchra*	Ecuador	RPEH191	AY461541	AY461557	AY847046	x	x	DQ061128
*V*. *quercifolia*	Bolivia	FTsn	JX092083	JX092046	JX091998	JX091957	JX091909	JX091868
*V*. *sphaerocarpa*	Colombia	SP6786	JX092079	JX092048	JX091993	JX091946	JX091911	JX091871
*V*. *sprucei*	Ecuador	AE8784	JX092085	x	JX092001	JX091960	-	JX091872
*V*. *stipulata*	Ecuador	RPEH55	AY461548	JX092049	AY847051	JX091961	JX091912	DQ061123
	Peru	IV17	x	**MT823598**	**MT823622**	**MT823652**	**MT823682**	**MT823712**
*V*. *weberbaueri*	Ecuador	RPEH10	AY461527	AY461573	x	JX091962	x	DQ061121
***Vasconcellea* sp.**	Peru	IV18	x	**MT823599**	**MT823623**	**MT823653**	**MT823683**	**MT823713**
	Peru	IV20	x	**MT823601**	**MT823625**	**MT823655**	**MT823685**	**MT823715**
	Peru	IV21	**MT808995**	**MT823602**	**MT823626**	**MT823656**	**MT823686**	**MT823716**
*Moringa oleifera* (Outgroups)	India	CFA2227	JX092069	KY697380	JX091981	JX091931	JX091889	DQ061137
*Moringa hildebrandtii* (Outgroups)	Madagascar	CFA2228	JX092068	JX092020	JX091980	JX091930	JX091888	JX091842

### Phylogenetic analyses

The phylogeny was based on concatenated data of the six molecular markers (65 sequences, Table 2). Selection of the best-fitting nucleotide substitution model was conducted using the program PartitionFinder [30] with six partitions (ITS, *matK*, *psbA-trnH*, *rbcL*, *rpl20-rps12*, *trnL-trnF*). The best partition strategy and model of sequence evolution were selected based on the Bayesian Information Criterion (BIC). The general time reversible nucleotide substitution model with a gamma distribution and a proportion of invariable sites (GTR + Γ + I) was selected for all partitions. Maximum likelihood (ML) analyses were conducted with the RAxML HPC-AVX program [31] implemented in the raxmlGUI 1.3.1 interface [32] using a GTRGAMMAI model with 1000 bootstrap replications. Bayesian inference (BI) was performed with MrBayes v. 3.2.6 software [33] using Metropolis-coupled MCMC and the GTR + Γ + I model. We conducted two runs each with four chains (three hot and one cold) for 10,000,000 generations, sampling trees every 1,000 generations. We plotted likelihood vs. generation using the Tracer Version v. 1.6 program [34] to reach a likelihood plateau and set the burn-in value.

### DNA-based species delimitation

We explored four different DNA-based delimitation methods using ITS, *matK*, *psbA-trnH*, *rbcL*, *rpl20-rps12*, and *trnL-trnF* data sets to assess species boundaries in *Vasconcellea*. Two of these DNA-based delimitation methods are based on genetic distance (statistical parsimony network analysis (SPN) [35] and automatic barcoding gap detection (ABGD) [36]) and two are based on coalescence (generalized mixed Yule coalescent method (GMYC) [37] and Bayesian phylogenetics and phylogeography (BPP) [38]).

For the SPN analyses of six markers, data sets were generated in TCS 1.21 [39] with a maximum connection probability set at 95% statistical confidence. The ABGD method was tested via a web interface (ABGD web, http://www.abi.snv.jussieu.fr/public/abgd/abgdweb.html). Before analysis, the model criteria were set as follows: variability (P) between 0.001 (Pmin) and 0.1 (Pmax), minimum gap width (X) of 1.0, Kimura-2-parameters and 50 screening steps.

To perform the GMYC delimitation method, an ultrametric tree was constructed in BEAST v.2.0.2 [40], relying on the uncorrelated lognormal relaxed clock, the GTR + Γ + I model, and a prior coalescent tree. Bayesian Markov chain Monte Carlo simulation was run for 50 million generations, and trees and parameters were sampled every 1000 generations. Log files were visualized in Tracer v.1.6 [34] for assessing the stationary state of parameters on the basis of the value of estimate-effective sample size (ESS). After removing 25% of trees as burn-in, the remaining trees were used to generate a single summarized tree in TreeAnnotator v.2.0.2 [40] as an input file for GMYC analyses. The GMYC analyses with a single threshold model were performed in R (R Development Core Team, http://www.R-project.org) under the ‘splits’ package using the ‘gmyc’ function (R-Forge, http://r-forge.r-project.org/projects/splits/).

To validate the outcomes of single locus species delimitation, a multilocus BPP was applied using the program BP&P v.2.0 [41, 42]. The six markers were used as inputs for BPP under the A11 model (A11: species delimitation = 1, species tree = 1). Specimens were *a priori* assigned to species based only on the minimum number of species from the results of the phylogenetic analysis. Five variables (ε1~ε5) were automatically fine-tuned following the instructions of BP&P [41]. The prior distribution of θ could have influenced the posterior probabilities for different models [41]. Analyses were run with three different prior combinations [43]. Each analysis was run five times to confirm consistency between runs. Two independent MCMC analyses were run for 100,000 generations with the ‘burn-in’ = 20,000.

### Nomenclature

The electronic version of this article in Portable Document Format (PDF) in a work with an ISSN or ISBN will represent a published work according to the International Code of Nomenclature for algae, fungi, and plants, and hence the new names contained in the electronic publication of a PLOS article are effectively published under that Code from the electronic edition alone, so there is no longer any need to provide printed copies.

In addition, new names contained in this work have been submitted to IPNI, from where they will be made available to the Global Names Index. The IPNI LSIDs can be resolved and the associated information viewed through any standard web browser by appending the LSID contained in this publication to the prefix http://ipni.org/. The online version of this work is archived and available from the following digital repositories: PubMed Central, LOCKSS.

## Results

### Molecular phylogeny

**Fig 2:**
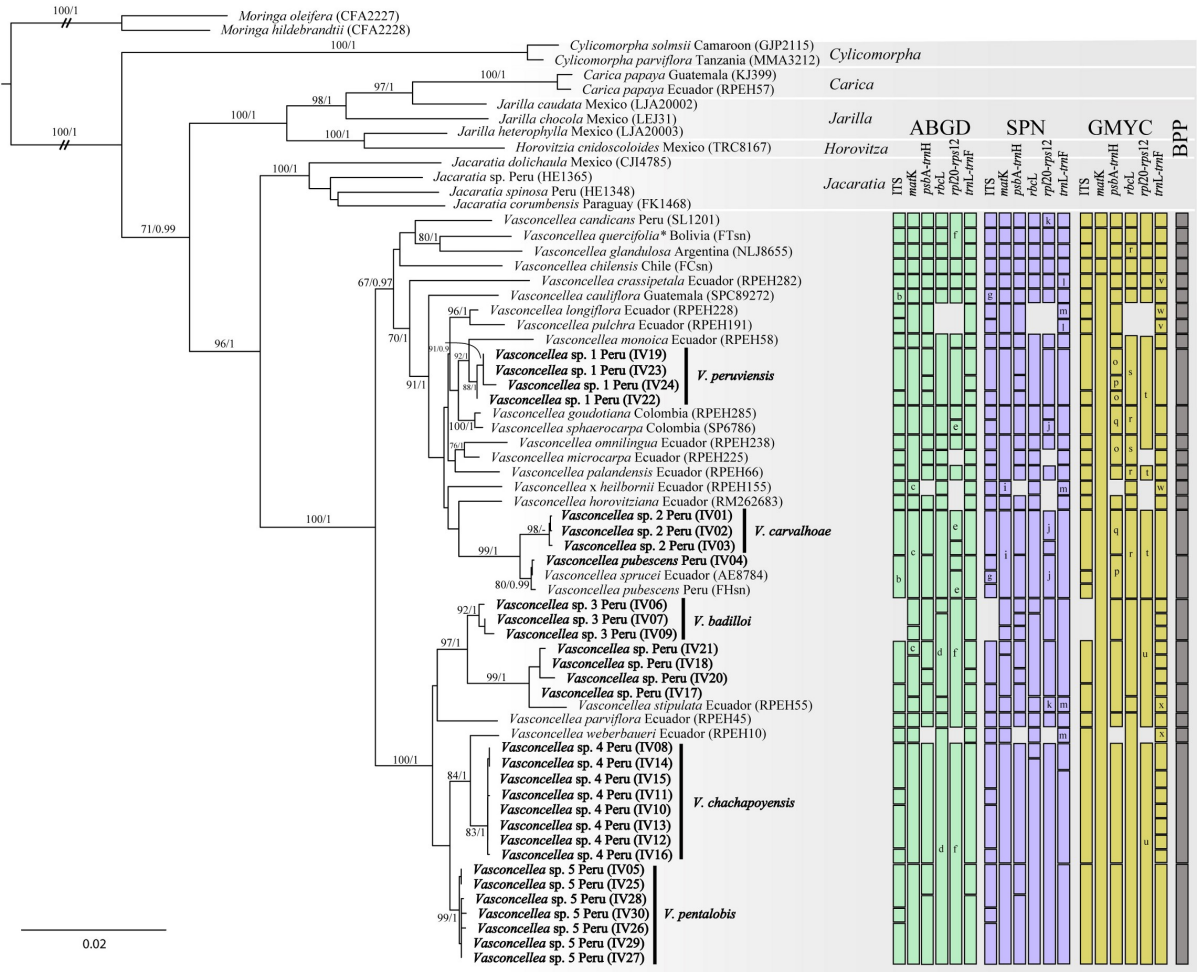
Phylogenetic tree based on maximum likelihood inference of combined *matK*, *rbcL*, *trnL*-*trnF*, *psbA*-*trnH*, *rpl20*-*prs12*, and ITS data. Value above branches = Maximum likelihood bootstrap values (BS)/Bayesian posterior probabilities (BI). Bars represent species delimitation results from ABGD-, SPN-, GMYC- and BPP-based algorithmic methods with six molecular markers. The scale bar indicates the number of nucleotide substitutions per site.

In the phylogeny of *Vasconcellea* species, the analysed data matrix included a total of 5208 base pairs (bp) (723 bp for ITS, 1089 bp for *matK*, 736 bp for *psb*A-*trn*H, 1362 bp for *rbcL*, 802 bp for *rpl*20-*prs*12, and 496 bp for *trn*L-*trn*F) from 65 individuals. Phylogenetic trees obtained from the ML and BI analyses confirmed the monophyly of the genus *Vasconcellea* and its sister relationship to the genus *Jacaratia* (Fig 2). Our multilocus phylogeny also molecularly confirmed 19 of the 21 recognized species of *Vasconcellea*, suggesting that *V*. *goudotiana* Triana & Planch and *V*. *sphaerocarpa* (García-Barr. & Hern. Cam.) V.M. Badillo as well as *V*. *pubescens* A. DC. and *V*. *sprucei* (V.M. Badillo) V.M. Badillo are conspecific (Fig 2). This multilocus phylogeny also distinguished the split of two evolutionary lineages in *Vasconcellea*. One of these lineages (BS/BI = 67/0.97) is composed of 18 species, including two unidentified species (*Vasconcellea* sp. 1 and *Vasconcellea* sp. 2). These unidentified species were determined to have sister relationship to *V*. *monoica* (Desf.) A. DC. and *V*. *pubescens*/*V*. *sprucei*, respectively. The second lineage in *Vasconcellea* is a well-supported clade (BS/BI = 100/1.0) composed of six species, including three unidentified species (*Vasconcellea* sp. 3, *Vasconcellea* sp. 4, and *Vasconcellea* sp. 5). *Vasconcellea* sp. 3 was found to have sister relationship to *V*. *stipulata* (V.M. Badillo) V.M. Badillo, and these two taxa were sisters to *V*. *parviflora* A. DC. *Vasconcellea* sp. 4 was found to have sister relationship to *V*. *weberbaueri* (Harms) V.M. Badillo, and these two taxa were sisters to *Vasconcellea* sp. 5.

Additionally, the tree topologies for two to four loci (S1*–*S3 Figs) and individual marker showed incongruence (S4 Fig). These trees showed slight topological differences in the evolutionary relationships among genera of Caricaceae but strong distinctiveness among species in the genus *Vasconcellea*. For instance, different gene trees (e.g., *trnL-trnF*, S4 Fig) showed distinguishing interspecific relationships among several species of *Vasconcellea*, indicating that these species share different common ancestors depending on the marker, which is understood under hybridization scenarios [44]. In addition, the genetic divergence comparisons of the six markers showed that there is not a minimum threshold (p-distance) for distinguishing genetic species in *Vasconcellea* (Table 3) confirming genetic discordance. For instance, *V*. *microcarpa* (Jacq.) A. DC. and *V*. *omnilingua* (V.M. Badillo) V.M. Badillo are identical when comparing *matK*, *psbA-trnH*, and *trnL-trnF*; but different when comparing ITS, *rbcL*, and *rpl20-rps12* (Table 3).

**Table 3:** Lowest genetic distance (p-distances) in percentage for species of *Vasconcellea* for six markers.

Taxa	Markers					
	ITS	*mat*K	*psb*A-*trn*H	*rbc*L	*rpl*20*-rps*12	*trn*L*-trn*F
*V*. *cauliflora—V*. *sprucei*	0.00	0.30	1.20	0.20	-	0.50
*V*. *microcarpa—V*. *omnilingua*	1.10	0.00	0.00	0.20	-	0.00
*V*. *pubescens—V*. *omnilingua*	0.40	0.20	0.00	0.30	0.50	0.50
*V*. *glandulosa—V*. *pubescens*	1.10	1.10	7.80	0.00	1.10	0.80
*V*. *parviflora—V*. *stipulata*	4.00	0.50	18.20	0.20	0.00	0.80
*V*. *badilloi—V*. *stipulata*	-	0.30	11.90	0.20	0.10	0.00
*V*. *chachapoyensis—V*. *weberbaueri*	1.10	0.60	-	0.00	-	0.30
*V*. *pentalobis—V*. *chachapoyensis*	1.10	0.30	6.00	0.00	0.00	0.50
*V*. *peruviensis—V*. *monoica*	0.40		7.80	0.00	0.40	0.80
*V*. *carvalhoae—V*. *pubescens*	0.36	0.00	-	0.00	0.00	0.00

### Species delimitation

The species-delimitation methods based on genetic distance (ABGD, SPN) and coalescence (GMYC, BPP) showed incongruent results for the six genes (Fig 2, Table 4). Among these methods, the highest number of species was delimited by the BPP analyses (25), whereas the most conservative results were obtained from GMYC (16 ± 10) (S5 and S6 Figs, S2 Table). Moreover, similar species numbers resulted from the ABGD (22 ± 6) and SPN (23 ± 5) analyses. The additional species delimitation by ABGD, BPP, and SPN was mainly due to the split of the clades composed of *V*. *pubescens*/*V*. *sprucei*, *V*. *stipulata*, *Vasconcellea* sp. 1, *Vasconcellea* sp. 2, *Vasconcellea* sp. 3, *Vasconcellea* sp. 4, and *Vasconcellea* sp. 5 (Fig 2). However, the split of these clades was not supported by the posterior probabilities obtained from BPP analyses (S3 Table). Although there were incongruent results in species number among different methods, the genetic distance methods (ABGD, SPN) and the multi-locus coalescent species validation (BPP) showed similar species numbers with those obtained in the phylogenetic analyses (Fig 2, Table 4). Regarding the six molecular makers, the highest number of species was delimited for the spacer ITS (27 ± 4) and the intergenic *trn*L-*trn*F (27 ± 6), whereas the lowest numbers were obtained for the genes *matK* (16 ± 10) and *rbcL* (17 ± 4) and the intergenic *rpl*20-*prs*12 (12 ± 5) (Table 4). These low species numbers were a consequence of the merging of several species that have similar sequences with null or very low genetic distance between these markers as a consequence of hybridization events (Table 3).

**Table 4:** Species number in *Vasconcellea* identified with DNA-based species-delimitations methods and phylogeny.

Taxa	Genetic Distance												Coalescence							Genealogical Concordance
	ABGD						SNP						GMYC							BPP
	ITS	*matK*	*psbA-trnH*	*rbcL*	*rpl20-rps12*	*trnL-trnF*	ITS	*matK*	*psbA-trnH*	*rbcL*	*rpl20-rps12*	*trnL-trnF*	ITS	*matK*	*psbA-trnH*	*rbcL*	*rpl20-rps12*	*trnL-trnF*	Multilocus	
***V*. *badilloi***	-	2	1	x	x	1	-	2	3	x	1	x	-	x	1	x	x	3	1	
*V*. *candicans*	1	1	1	1	x	1	1	1	1	1	x	1	1	1	1	1	x	1	1	
*V*. *cauliflora*	x	1	1	1	1	1	x	1	1	1	1	1	1	x	1	1	1	1	1	
***V*. *carvalhoae***	1	x	1	x	x	1	1	x	1	x	x	x	x	x	x	x	x	x	1	
***V*. *chachapoyensis***	4	1	1	x	x	1	4	1	1	x	x	2	x	x	1	x	x	7	1	
*V*. *chilensis*	1	1	1	1	1	1	1	1	1	1	1	1	1	1	1	1	1	1	1	
*V*. *crassipetala*	1	1	1	1	1	1	1	1	1	1	1	x	1	x	1	1	1	x	1	
*V*. *pubescens / V*. *sprucei*	x	x	1	x	x	1	x	x	1	x	x	x	x	x	x	x	x	x	1	
*V*. *glandulosa*	1	1	1	1	x	1	1	1	1	1	1	1	1	x	1	x	1	1	1	
*V*. *goudotiana / V*. *sphaerocarpa*	1	1	1	1	x	1	2	1	1	1	x	1	1	x	x	x	x	1	1	
*V*. *x heilbornii*	x	x	-	1	-	1	x	x	-	1	-	x	x	x	-	1	-	x	1	
*V*. *horovitziana*	1	1	1	1	-	1	1	1	1	1	-	1	1	x	1	1	-	1	1	
*V*. *longiflora*	1	x	x	-	-	x	1	x	x	-	-	x	1	x	x	-	-	x	1	
*V*. *microcarpa*	1	x	1	1	-	1	1	x	1	1	-	1	1	x	x	x	-	1	1	
*V*. *monoica*	1	1	1	x	1	1	1	1	1	x	1	1	x	x	1	x	x	1	1	
*V*. *omnilingua*	1	x	1	1	1	1	1	x	1	1	1	1	1	x	x	x	x	1	1	
*V*. *palandensis*	1	x	1	1	1	1	1	x	1	1	1	1	1	x	1	x	x	1	1	
*V*. *parviflora*	1	1	1	1	x	1	1	1	1	1	1	1	1	x	1	x	x	1	1	
***V*. *pentalobis***	3	1	2	x	x	2	3	1	2	x	x	1	1	x	1	x	x	1	1	
***V*. *peruviensis***	2	1	3	x	1	3	2	1	3	x	1	1	x	x	x	x	x	1	1	
*V*. *pulchra*	1	x	x	-	-	x	1	x	x	-	-	x	1	x	x	-	-	x	1	
*V*. *quercifolia*	1	1	1	1	x	1	1	1	1	1	1	1	1	x	1	1	x	1	1	
*V*. *stipulata*	1	x	1	x	x	1	1	x	1	x	x	x	1	x	x	x	x	x	1	
*V*. *weberbaueri*	1	1	-	x	-	1	1	1	-	1	-	x	x	x	-	x	-	x	1	
***Vasconcellea* sp.**	x	x	2	x	x	2	x	x	2	x	x	x	x	x	x	x	x	3	1	
*Total*	**28**	**21**	**26**	**18**	**12**	**28**	**31**	**22**	**28**	**21**	**17**	**20**	**23**	**4**	**18**	**12**	**7**	**32**	**28**	

### Taxonomic treatment

Our molecular analyses revealed that the five unidentified species of *Vasconcellea* were strongly supported as distinct entities by BPP and multilocus phylogeny and confirmed by ABGD (ITS, *psb*A-*trn*H, *trn*L-*trn*F) and SPN (ITS, *psb*A-*trn*H). This molecular examination of *Vasconcellea* taxa based on an integrative approach justifies the proposal of the following five taxa as new species: *V*. *badilloi* sp. nov. (= *Vasconcellea* sp. 3), *V*. *carvalhoae* sp. nov. (= *Vasconcellea* sp. 2), *V*. *chachapoyensis* sp. nov. (= *Vasconcellea* sp. 4), *V*. *pentalobis* sp. nov. (= *Vasconcellea* sp. 5), and *V*. *peruviensis* sp. nov. (= *Vasconcellea* sp. 1)

**Fig 3:**
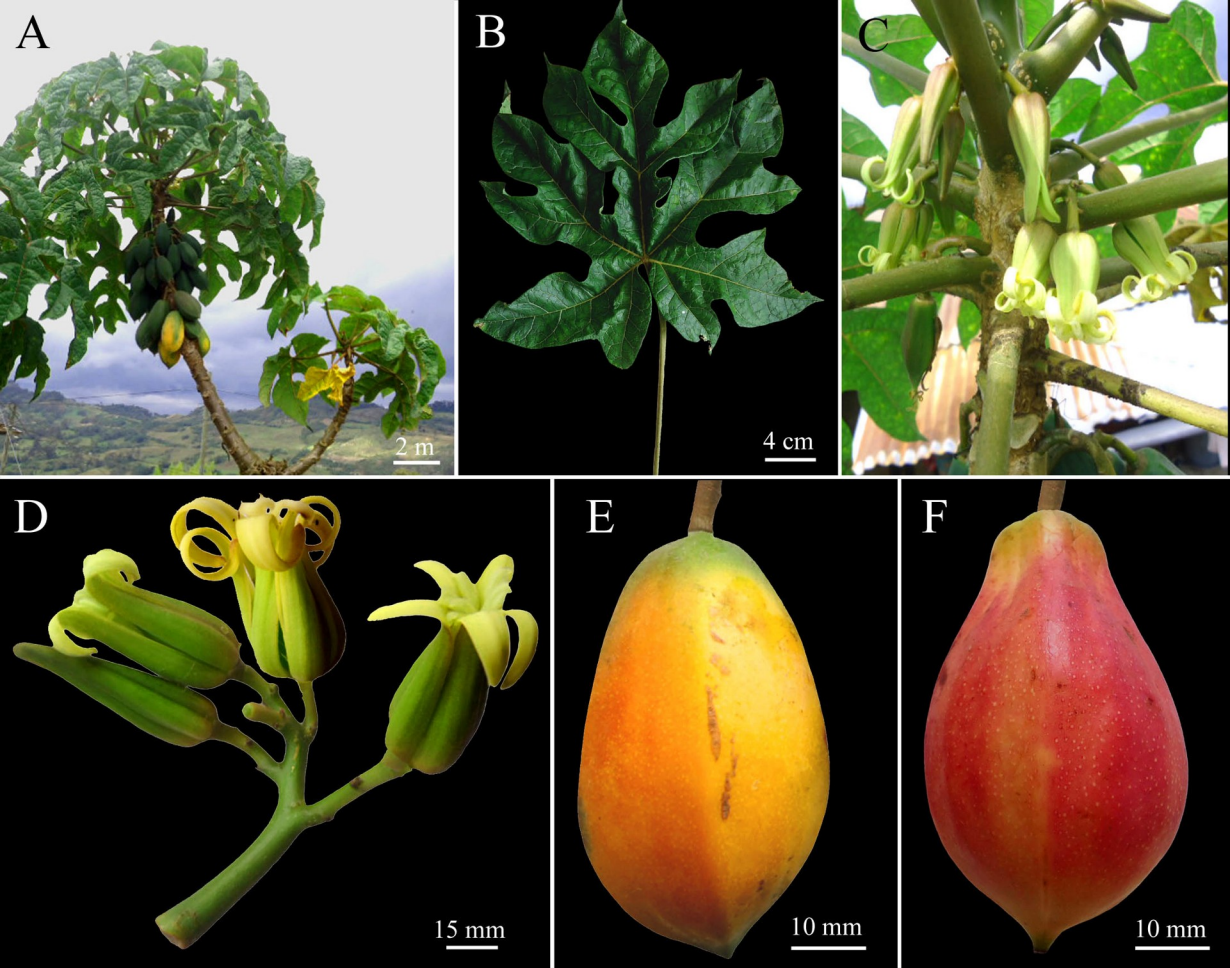
Morphology of *Vasconcellea badilloi* sp. nov. (CHAX224). **A,** Habit. **B,** Palmately compound leaf. **C,** Axillary flowers. **D,** Female inflorescence. **E, F,** Mature fruit.

***Vasconcellea badilloi*** D. Tineo & D.E. Bustam., **sp. nov.** (Fig 3)

[urn:lsid:ipni.org:names: 77212844–1]

#### Holotype

Peru, Amazonas, Prov. Bongará, Dist. Pomacochas, 5°48'53''S, 77°57'24"W, 2280 m a.s.l., 13 Sep. 2018, *D*. *Tineo IV06* (holotype, CHAX224).

#### Diagnosis

Dioecious tree to 6 m tall having as a distinguishing feature its yellow-orange to pink ovoid berries. Species very similar morphologically to *V*. *carvalhoae* and *V*. *pubescens* but differing in the phylogenetic relationship with these species. Furthermore, *V*. *badilloi* is distinguished genetically from *V*. *pubescens* (1.2% for *matK*, 8.4% for *psb*A-*trn*H, 0.5% for *rbcL*, 1.3% for *rpl*20-*rps*12, 0.4% for *trn*L-*trn*F) and *V*. *carvalhoae* (1.2% for *matK*, 8.4% for *psb*A-*trn*H, 0.5 for *rbcL*, 1.3% for *rpl*20-*rps*12, 0.3% for *trn*L-*trn*F).

#### Description

Dioecious tree to 6 m tall (Fig 3A); bark light brown, covered with leaf scars; stipules absent. Latex white milky. Leaves roughly textured, alternate, crowded at top of tree, palmately compound (Fig 3B); petiole to 75 cm long; leaflets 5 to 7, glabrous and bright green above, lighter green below; 4 to 5 basal leaflets entire 18–30 × 7–9 cm, widely elliptic to widely ovate, base acute, apex acuminate; central leaflet trilobed, 2 lateral lobes 12–15 × 3.5–5 cm, elliptic to ovate, apex acuminate, central lobe 20–35 × 4–8 cm, elliptic to ovate, base acute, apex acuminate; veins raised beneath, primary vein often reddish. Female inflorescences axillary (Fig 3C and 3D), cymose, 4 to 5 cm long; peduncle 1–2 cm long, 3 mm diam.; pedicels 2–3 mm long, with a few small bracts 1 mm long. Female flowers 5-merous. Sepals green, triangular, 2–3 × 1–2 mm. Petals green-yellowish outside, green inside, free, oblong-obtuse, 25–35 × 5–7 mm, apex obtuse. Sepals and petals alternate. Ovary superior, 5-locular, 5-angular, 9–18 × 5–8 mm, attenuate towards apex; numerous anatropous ovules on parietal placentas; style 4–5 mm long; stigmas 5, 5–7 mm long, apically often split in 2 ends of 2–3.5 mm each. Male inflorescences were not found in this study. Fruit an ovoid berry, yellow-orange to pink, base rounded to emarginate, apex acute, 60–75 × 4–5.5 mm; pericarp 4–5 mm thick; pedicel of fruit 9–12 × 4–5 mm (Fig 3E and 3F). Seeds dark brown, 7–8 × 4–5 mm, ellipsoidal, sclerotesta with conical protuberances, each seed surrounded by a gelatinous sarcotesta, seeds arranged in 5 groups surrounded by yellowish pulp. Strong aroma. The sugar content varies from 6 to 7°Brix.

#### Etymology

The specific epithet '*badilloi*' honours Victor M. Badillo for his pioneering and valuable contributions to the understanding of Caricaceae in South America, especially in the genus *Vasconcellea*.

#### Ecology and distribution

The species is known from the area around Pomacochas (5°48'53''S 77°57'24"W) in the province of Bongará (Amazonas, Peru) and around Quinjalca (6°05'33"S 77°40'39"W) in the province of Chachapoyas (Amazonas, Peru). It is found in the wild in wet premontane to montane forests at 1300–3200 m elevation. Plants are cultivated.

#### Specimens examined

Peru, Amazonas, Prov. Bongará, Dist. Cuchulia, 5°59'44''S, 77°58'30'W', 1386 m a.s.l., 13 Sep. 2018, *D*. *Tineo IV07* (CHAX225); Peru, Amazonas, Prov. Chachapoyas, Dist. Quinjalca, 6°05'33"S, 77°40'39"W, 3143 m a.s.l., 20 Sep. 2018, *D*. *Tineo IV09* (CHAX226).

#### Remarks

*Vasconcellea badilloi* highly resembles *V*. *carvalhoae* and *V*. *pubescens* in morphology, growing in sympatry. However, *V*. *badilloi* is distinguished by its yellow-orange to pink ovoid berries. *V*. *badilloi* is also distinguished from *V*. *pubescens* by the lack of pubescence on the leaves. Furthermore, *V*. *badilloi* is distantly related to *V*. *carvalhoae* and *V*. *pubescens* with multilocus phylogeny. In addition, although *V*. *badilloi* is phylogenetically closely related to *V*. *stipulata*, *V*. *badilloi* is distinguished from *V*. *stipulata* by the lack of stipules (S4 Table).

**Fig 4:**
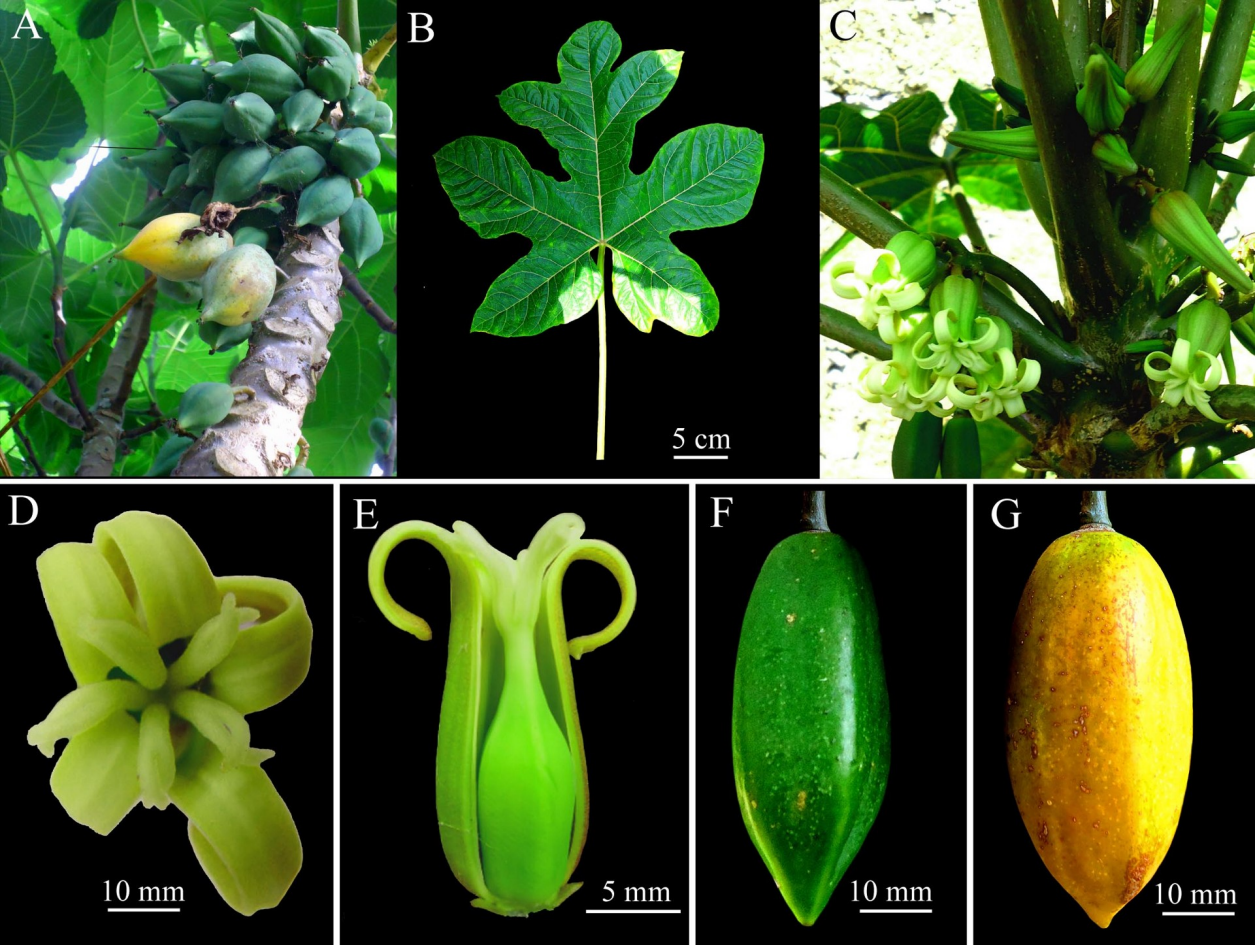
Morphology of *Vasconcellea carvalhoae* (CHAX227). **A,** Habit. **B,** Palmately compound leaf. **C,.** Female inflorescence. **D, E,** Female flowers with stigmas split into 2–3 ends (D) and superior ovaries (E). **F,** Immature fruit. **G,** Mature fruit.

***Vasconcellea carvalhoae*** D. Tineo & D.E. Bustam., **sp. nov**. (Fig 4)

[urn:lsid:ipni.org:names: 77212845–1]

#### Holotype

Peru, Amazonas, Prov. Bongará, Dist. Pomacochas, 5°49'45"S, 77°58'12"W, 2232 m a.s.l., 02 Oct. 2018, *D*. *Tineo IV01* (holotype, CHAX227).

#### Diagnosis

Dioecious tree to 4 m tall that is very similar morphologically to *V*. *sprucei*/*V*. *pubescens*, but differing in the sister phylogenetic relationship with these species. The sequence divergence between *V*. *carvalhoae* and *V*. *sprucei*/*V*. *pubescens* is 0.36% for the ITS region.

#### Description

Dioecious tree to 4 m tall; bark light brown, covered with leaf scars. Latex white milky (Fig 4A). Leaves membranaceus, alternate, crowded at top of tree, palmately compound; petiole to 60 cm long (Fig 4B); leaflets 5, glabrous and bright green above, lighter green below; 4 basal leaflets entire, 20–30 × 10–15 cm, widely elliptic to widely ovate, base acute, apex acute to rounded; central leaflet trilobed, 2 lateral lobes 17–19 × 7–10 cm, widely elliptic to widely ovate, apex acute to rounded, central lobe 35–40 × 10–12 cm, widely elliptic to widely ovate, apex acute to rounded; veins raised beneath, primary vein often reddish. Female inflorescences axillary (Fig 4C), cymose, many-flowered, to 16 cm long; peduncle 5–7 cm long, 4–7 mm diam.; pedicels 2–3 cm long, with a few small bracts 1 mm long. Female flowers 5-merous. Sepals green, triangular, 2–3.5 × 1.5–2 mm. Petals green-yellow outside, green inside, free, oblong-obtuse, 30–40 × 5.5–7 mm, apex obtuse. Sepals and petals alternate. Ovary superior, 5-locular, 5-angular, 10–20 × 6–9 mm, attenuate towards apex; numerous anatropous ovules on parietal placentas; style 3–4 mm long; stigmas 5, 5–8 mm long, apically often split in 2 ends of 3–5 mm each (Fig 4D and 4E). Fruit an ovoid berry (Fig 4F and 4G), yellow, base rounded to emarginate, apex acute, 60–80 × 42–56 mm; pericarp 4–5.5 mm thick; pedicel of fruit 10–17 × 4.5–5 mm. Seeds light brown, 5–7 × 3–5 mm, ellipsoidal, sclerotesta with numerous conical protuberances, each seed surrounded by a gelatinous sarcotesta, seeds arranged in 5 groups surrounded by yellow-white pulp. The sugar content varies from 7.5 to 8°Brix.

#### Etymology

The specific epithet ‘*carvalhoae’* honours Fernanda A. Carvalho for her valuable contributions to the understanding of the Caricaceae in the bioinformatics era.

#### Ecology and distribution

The species is known from the area around Pomacochas (5°49'45"S 77°58'12"W) in the province of Bongará, Amazonas, Peru. It is found in the wild in montane areas at 2236 m elevation. Plants not cultivated.

#### Specimens examined

Peru, Amazonas, Prov. Bongará, Dist. Pomacochas, 5°49'08.7"S, 77°57'39.3"W, 2263 m a.s.l., 02 Oct. 2018, *D*. *Tineo IV02* (CHAX228); Peru, Amazonas, Prov. Bongará, Dist. Pomacochas, 5°49'37"S, 77°58'01"W, 2236 m a.s.l., 02 Oct. 2018, *D*. *Tineo IV03* (CHAX229).

#### Remarks

*Vasconcellea carvalhoae* is highly similar in morphology to *V*. *sprucei*/*V*. *pubescens*, growing in sympatry. However, these species are distinguished by their elongated ovoid berry and lack of pubescence (S4 Table). Phylogenetically, *V*. *carvalhoae* is also a sister to the clade composed of *V*. *sprucei*/*V*. *pubescens*. They are genetically different species based on a 0.36% divergence in the ITS region.

**Fig 5:**
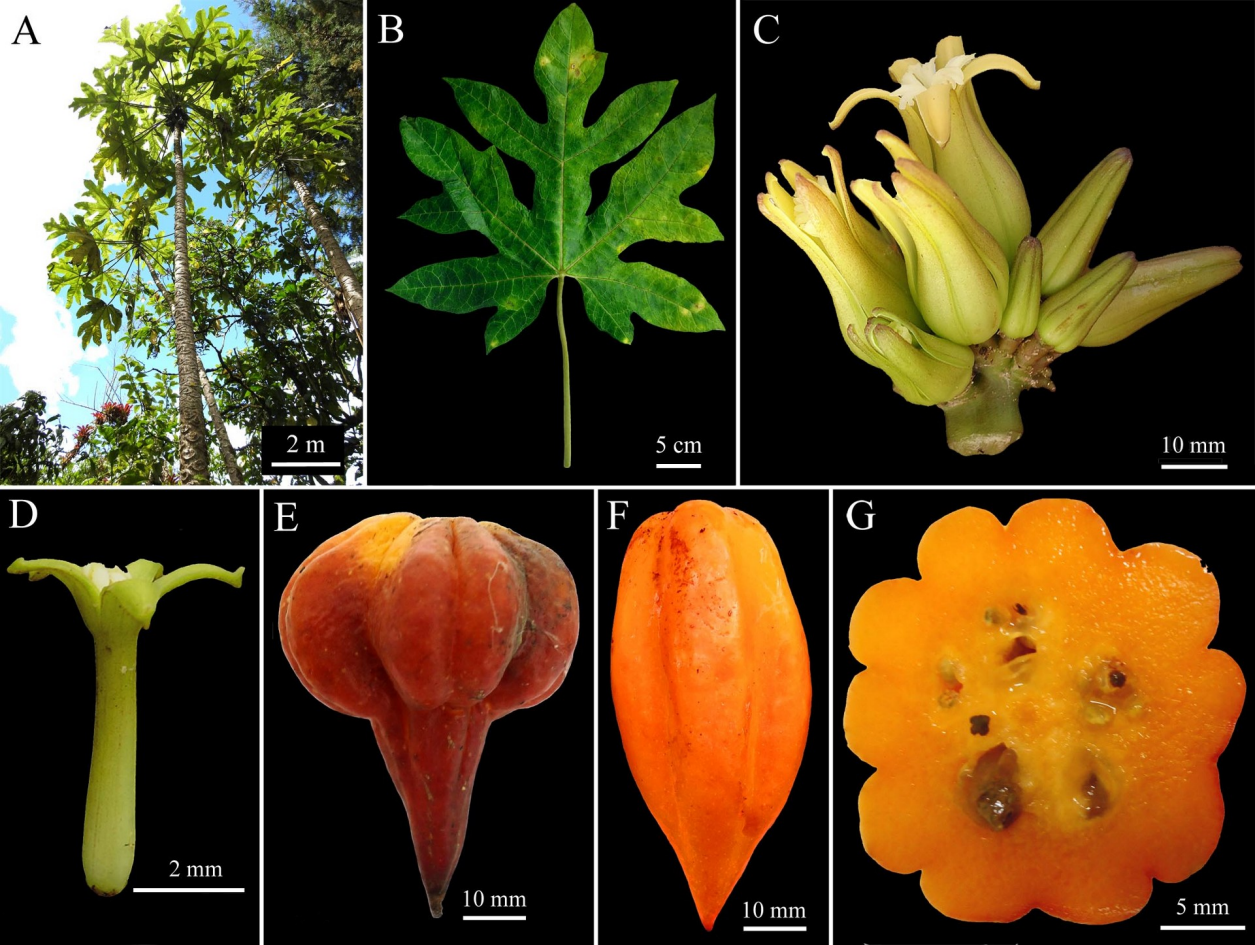
Morphology of *Vasconcellea chachapoyensis* sp. nov. (CHAX235). **A,** Habit. **B,** Palmately compound leaf. **C,** Female inflorescence. **D,** Male flower. **E, F,** Mature fruits with acuminate apex. **G,** 5-locular ovary.

***Vasconcellea chachapoyensis*** D. Tineo & D.E. Bustam., **sp. nov.** (Fig 5)

[urn:lsid:ipni.org:names: 77212846–1]

#### Holotype

Peru, Amazonas, Prov. Chachapoyas, Dist. Asunción Goncha, 6°01'56.6"S, 77°42'37.1"W, 2821 m a.s.l., 20 Sep. 2018, *D*. *Tineo IV14* (holotype, CHAX235).

#### Diagnosis

Dioecious tree to 12 m tall having ovoid berries with acuminate apex as distinguishing features. Species very similar morphologically to *V*. *weberbaueri*, but differing in the larger inflorescence and the wider leaflets at the base and in the sister phylogenetic relationship with this species. The sequence divergence between *V*. *chachapoyensis* and *V*. *weberbaueri* is 1.1% for the ITS, 0.6% for *matK*, and 0.3% for *trn*L-*trn*F.

#### Description

Dioecious tree to 12 m tall (Fig 5A); bark light brown, covered with prominent leaf scars; stipules absent. Latex white milky. Leaves membranaceus, alternate, crowded at top of tree, palmately compound, deeply lobe (Fig 5B); petiole 50 to 70 cm long; leaflets 5 to 7, glabrous and bright green above, lighter green below with purple red stripes and stipules in the veins; 4 to 7 basal leaflets entire, 9.5–30.2 × 6.6–12.4 cm, widely elliptic to ovate, base obtuse, apex acuminate; central leaflet trilobed, 2 lateral lobes 12–18.5 × 4.5–7.6 cm, widely elliptic to ovate, apex acuminate, central lobe 25–38 × 8–10.7 cm, elliptic to ovate, base acute, apex acuminate (Fig 5B). Female inflorescences axillary (Fig 5C), cymose, few-flowered, to 9 cm long; peduncle 3–4 cm long, 4–5 mm diam.; pedicels 5–15 mm long, with a few small bracts 1.5 mm long. Female flowers 5-merous. Sepals greenish, triangular, 3–4 × 2–3 mm. Petals green-yellowish, green inside, free, oblong-triangular, 31–42 × 6–9 mm, apex obtuse. Sepals and petals alternate. Ovary superior, 5-locular, 5-angular, 6–8 × 3–4 mm, attenuate towards apex; numerous anatropous ovules on parietal placentas; style 5–8 mm long; stigmas 5, 7–12 mm long, short, apically often split in 2–4 ends of 2–4 mm each. Male inflorescences axillary, many-flowered panicles, to 25–35 cm long, pubescent; peduncle 15–20 cm long, to 4 mm diam.; lateral branches 3–6 cm long; pedicels 2–6 mm long, with a few small bracts to 1 mm long. Male flowers 5-merous (Fig 5D). Sepals brown, triangular, 2–3 × 1–2 mm. Corolla green-yellow; tube 16–23 mm long, 3–4 mm wide at base, 1.5–2 mm wide in the middle, 2.5–4 mm wide at apex; lobes oblong-lanceolate, 14–20 × 2–3 mm, apex acute. Sepals and petals alternate. Stamens 10, in 2 series, attached at apex of corolla tube, versatile, 2 thecae each, opening with longitudinal slits, introrse; upper stamens with loosely pilose filaments 2–2.4 mm long, anther glabrous, 2–2.5 mm long, anther connective; lower stamens with filament 1–1.5 mm long, anther glabrous, 2–3 mm long. Rudimentary gynoecium 9 to 11 mm long. Fruit an ovoid berry, yellow-orange, base emarginate, apex acuminate, 75–85 × 35–45 mm; pericarp 12–15 mm thick; pedicel of fruit 3–4 × 1–3 mm (Fig 5E and 5F). Seeds light brown, 4–5 × 3–4 mm, ellipsoidal, sclerotesta with numerous conical protuberances, each seed surrounded by a gelatinous sarcotesta, seeds arranged in 5 groups surrounded by orange pulp. Starry central cavity (Fig 5G). The sugar content varies from 6 to 6.5°Brix.

#### Etymology

The specific epithet ‘*chachapoyensis’* is derived from the province where the samples were collected.

#### Ecology and distribution

The species is known from the area around Chachapoyas (6°01'56.6"S 77°42'37.1"W) in the Region Amazonas, Peru. It is found in the wild in humid montane forest at 2400–3800 m elevation. Plants are not cultivated.

#### Specimens examined

Peru, Amazonas, Prov. Chachapoyas, Dist. Quinjalca, 6°05'30.4"S, 77°40'30.4"W, 3130 m a.s.l., 20 Sep. 2018, *D*. *Tineo IV08* (CHAX230); Peru, Amazonas, Prov. Chachapoyas, Dist. Quinjalca 6°05'25''S, 77°40'46''W, 3150 m a.s.l., 20 Sep. 2018, *D*. *Tineo IV15* (CHAX236); Peru, Amazonas, Prov. Chachapoyas, Dist. Olleros, 6°03'07''S, 77°38'54''W, 3041 m a.s.l., 20 Sep. 2018, *D*. *Tineo IV11* (CHAX232); Peru, Amazonas, Prov. Chachapoyas, Dist. Olleros, 6°03'13.2"S, 77°38'47.3"W, 3031 m a.s.l., 20 Sep. 2018, *D*. *Tineo IV12* (CHAX233); Peru, Amazonas, Prov. Chachapoyas, Dist. Granada, 6°06'12''S, 77°37'47''W, 2996 m a.s.l., 20 Sep. 2018, *D*. *Tineo IV10* (CHAX231); Peru, Amazonas, Prov. Chachapoyas, Dist. Granada, 6°06'10''S, 77°37'39''W, 3017 m a.s.l., 20 Sep. 2018, *D*. *Tineo IV13* (CHAX234); Peru, Amazonas, Prov. Chachapoyas, Dist. Molinopampa, 6°16'59.2''S, 77°33'31.7"W, 2200 m a.s.l., 25 Sep. 2018, *D*. *Tineo IV16* (CHAX237).

#### Remarks

*Vasconcellea chachapoyensis* is highly similar in morphology to *V*. *weberbaueri*, but it is distinguished by its larger inflorescence and wider leaflets at the base (S4 Table). Phylogenetically, *V*. *chachapoyensis* is also a closely related species to *V*. *weberbaueri*. However, these two species are genetically different at the ITS (1.1%), *matK* (0.6%), and *trn*L-*trn*F (0.3%) loci. Additionally, *V*. *chachapoyensis* grows in sympatry with *V*. *pentalobis*.

**Fig 6:**
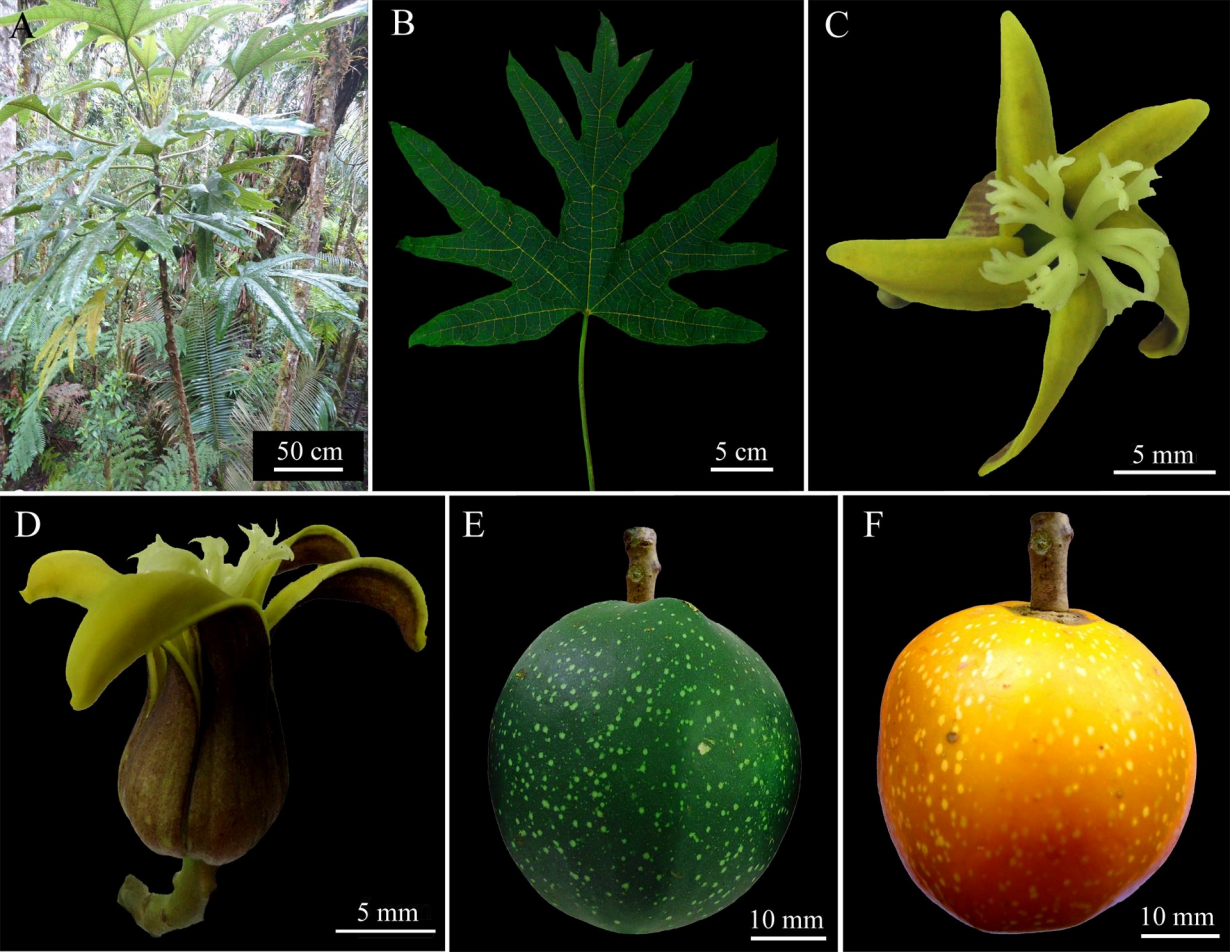
Morphology of *Vasconcellea pentalobis* sp. nov. (CHAX238). **A,** Habit. **B,**. Palmately compound leaf with four basal leaflets and a central pentalobed leafleft. **C,** Female flower with stigmas split in 2–3 ends. **D,** Dextrorotatory female flower. **E,** Immature fruit. **F,** Mature fruit.

***Vasconcellea pentalobis*** D. Tineo & D.E. Bustam., **sp. nov.** (Fig 6)

[urn:lsid:ipni.org:names: 77212847–1]

#### Holotype

Peru, Amazonas, Prov. Chachapoyas, Dist. Molinopamapa, 6°14'49''S 77°32'50''W, 2297 m a.s.l., 05 May. 2018, *D*. *Tineo IV05* (holotype, CHAX238).

#### Diagnosis

Dioecious tree to 8 m tall that is morphologically distinguished from any other species of *Vasconcellea* by the pentalobed central leaflet (four laterals and one central), dextrorotatory female flowers, and globose berries.

#### Description

Dioecious tree to 8 m tall (Fig 6A); bark light brown, covered with leaf scars; stipules absent. Latex white milky. Leaves membranaceus, alternate, slightly crowded at top of tree, palmately compound (Fig 6B); petiole to 40 to 50 cm long; leaflets 5 to 6, glabrous and bright green above, lighter green below with primary veins often green; usually 4 basal leaflets entire 30–39 × 10–13.5 cm, elliptic, base acute, apex acute; central leaflet pentalobed, 4 lateral lobes 15–35 × 4–8 cm, elliptic, apex acute, central lobe 10–18 × 3.5–6 cm, elliptic to ovate, base acute, apex acuminate (Fig 6B). Female inflorescences axillary, cymose, few-flowered, to 9 cm long, dextrorotatory (Fig 6C and 6D); peduncle 3.5–5 cm long, 3–4.5 mm diam.; pedicels 4.5–7.5 mm long, with a few small bracts 0.5–1.7 mm long. Female flowers 5-merous. Sepals green-reddish, triangular, 2–3 × 1–2 mm. Petals yellowish, free, oblong-triangular, 40–50 × 7–9 mm, apex obtuse. Sepals and petals alternate. Ovary superior, 5-locular, 5-angular, 9–15 × 6–12 mm, attenuate towards apex; numerous anatropous ovules on parietal placentas style 4–6 mm long stigmas 5, 5–7 mm long, apically often split in 2–3 ends of 0.5–2 mm each (Fig 6C). Male inflorescences were not found in this study. Fruit a globose berry, yellow-greenish with white dots, base slightly flattened, apex rounded, 50–65 × 50–60 mm; pericarp 11–15 mm thick; pedicel of fruit 6–8 × 3–5 mm (Fig 6E and 6F). Seeds light brown, 4–5 × 3.5–4 mm, ellipsoidal, sclerotesta with small and numerous conical protuberances, each seed surrounded by a gelatinous sarcotesta, arranged in 5 groups surrounded by intense orange pulp. Semistarred central cavity. The sugar content varies from 7.5 to 8°Brix.

#### Etymology

The specific epithet ‘*pentalobis’* refers to the diagnostic feature of the central leaflets that is composed of five lobes (four laterals and one central lobe).

#### Ecology and distribution

The species is known from the area around Chachapoyas (6°14'49''S 77°32'50''W) in the Region Amazonas, Peru. It is found in the wild in humid montane forest at 1600–2800 m elevation. Plants are not cultivated.

#### Specimens examined

Peru, Amazonas, Prov. Chachapoyas, Dist. Molinopampa, 6°15'35''S, 77°32'45''W, 2406 m a.s.l., 5 May. 2018, *D*. *Tineo IV25* (CHAX239); Peru, Amazonas, Prov. Chachapoyas, Dist. Molinopampa, 6°20'08"S, 77°31'49''W, 2385 m a.s.l., 20 Nov. 2019, *D*. *Tineo IV28* (CHAX242); Peru, Amazonas, Prov. Chachapoyas, Dist. Molinopampa, 6°20'41''S, 77°31'17''W, 2321 m.a.s.l., 20 Nov. 2019, *D*. *Tineo IV29* (CHAX243); Peru, Amazonas, Prov. Chachapoyas, Dist. Molinopampa, 6°18'59''S, 77°33'21''W, 2659 m a.s.l., 20 Nov. 2019, *D*. *Tineo IV30* (CHAX244); Peru, Amazonas, Prov. Chachapoyas, Dist. La jalca, 6°28'25''S, 77°42'13''W, 2523 m a.s.l., 15 Nov. 2019, *E*. *Huaman IV26* (CHAX240); Peru, Amazonas, Prov. Chachapoyas, Dist. La Jalca, 6°28'25''S, 77°41'51''W, 2342 m a.s.l., 15 Nov. 2019, *E*. *Huaman IV27* (CHAX241).

#### Remarks

*Vasconcellea pentalobis* is morphologically distinguished from any other species of *Vasconcellea* by the pentalobed central leaflet (four laterals and one central), dextrorotatory female flowers, and globose berries (S4 Table). *V*. *pentalobis* is sister to the clade composed of *V*. *chachapoyensis* and *V*. *weberbaueri* with our multilocus phylogeny. Additionally, *V*. *pentalobis* grows in sympatry with *V*. *chachapoyensis*.The genetic divergence between *V*. *pentalobis* and *V*. *chachapoyensis* is 1.1% for ITS, 0.3% for *matK*, 3.0% for *psb*A-*trn*H, and 0.6% for *trn*L-*trn*F, whereas that between *V*. *pentalobis* and *V*. *weberbaueri* is 1.4% for the ITS, 0.3% for *matK* and 0.3% for *trn*L-*trn*F

**Fig 7:**
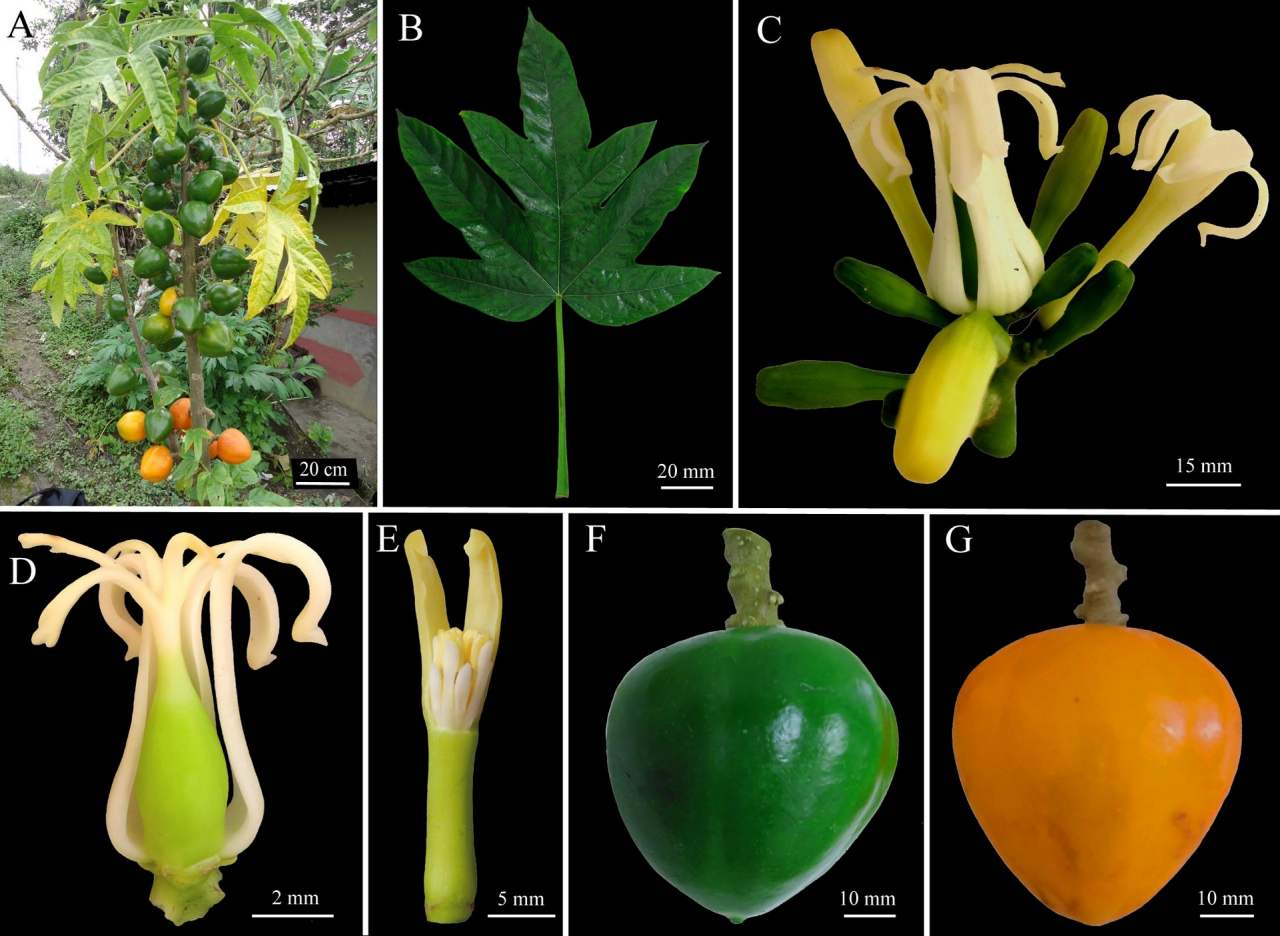
Morphology of *Vasconcellea peruviensis* (CHAX245). **A,** Habit. **B,** Palmately compound leaf. **C,** Female inflorescence. **D,** Pistillate flower. **E,** Staminate flower. **F,** Immature fruit. **G,** Mature fruit.

***Vasconcellea peruviensis*** D. Tineo & D.E. Bustam., **sp. nov.** (Fig 7)

[urn:lsid:ipni.org:names: 77212848–1]

#### Holotype

Peru, Amazonas, Prov. Utcubamba, Dist. Cajaruro, 5°40'04"S 78°20'17''W, 1538 m.a.s.l., 19 Oct. 2018, *D*. *Tineo IV23* (holotype, CHAX247).

#### Diagnosis

Monoecious tree to 4 m tall that is very similar morphologically to *V*. *monoica*, but differing by its globose berries with acute apices and in the sister phylogenetic relationship with this species. The sequence divergence between *V*. *peruviensis* and *V*. *monoica* is 0.4% for the ITS, 0.3–0.4% for *matK*, 4.6–5% for *psb*A-*trn*H, 0.3–0.4% for *rpl*20-*rps*12, and 0.6–0.8% for *trn*L-*trn*F.

#### Description

Monoecious tree to 4 m tall (Fig 7A); bark light brown and glabrous; slightly covered with leaf scars. Latex white milky. Leaves membranaceus, alternate, crowded at top of the stem, palmately compound (Fig 7B); petiole to 35 cm long; leaflets 5, glabrous and bright green above, lighter green below; 4 basal leaflets entire, 12–16 × 4–7 cm elliptic, base acute, apex acute to acuminate; central leaflet trilobed, 2 lateral lobes 9–12 × 3–5 cm, elliptic, apex acuminate, central lobe 20–25 × 5–7 cm, elliptic to ovate, base acute, apex acuminate; elevated veins below, primary vein green-reddish. Female and male inflorescences axillary grouped in few-flowered panicles, to 5 cm long; peduncle 1–3 cm long, to 2–3 mm diam.; lateral branches 1–3 cm long; pedicels 1–3 mm long, with a few small bracts to 1 mm long (Fig 7C). Female flowers 5-merous (Fig 7D). Sepals green, triangular, 1.5–2 × 0.5–1 mm. Petals yellow-white outside and inside, free, oblong-triangular, 33–45 × 5–7 mm, apex obtuse. Sepals and petals alternate. Ovary superior, 5-locular, 5-angular, 9–15 × 7–10 mm, attenuate towards apex; numerous anatropous ovules on parietal placentas; style 3–4.5 mm long; stigmas 5, 7–12 mm long, apically often split in 2 ends of 3–4 mm each. Male flowers 5-merous (Fig 7E). Sepals green, triangular, 1–2 × 0.5–1 mm. Corolla yellow-white; tube 18–22 mm long, 2–4 mm wide at base, 1.5–3 mm wide in the middle, 2–3.5 mm wide at apex; lobes oblong-lanceolate, 16–23 × 2–5 mm, apex acute. Sepals and petals alternate. Stamens 10, in 2 series, attached at the apex of corolla tube, versatile, 2 thecae each, opening with longitudinal slits, introrse; upper stamens with pilose filaments 1.5–2 mm long, anther glabrous, 2–3 mm long; lower stamens with filament 1 mm long, anther glabrous, 2–3 mm long, anther connective prolonged for 1 mm. Rudimentary gynoecium 6–9 mm long. Fruit a globose berry, yellow-orange, base rounded, apex acute, 10–18 to 6–7 cm; pericarp 4–5 mm thick; pedicel of fruit 6–9 × 4–5 mm (Fig 7F and 7G). Seeds dark brown, 6–8 × 4–5.5 mm, ellipsoidal, sclerotesta with numerous large and conical protuberances, each seed surrounded by a gelatinous sarcotesta, seeds arranged in 5 groups surrounded by yellow-white pulp. Superficial or low depression ridges. The sugar content varies from 4.5 to 5°Brix.

#### Etymology

The specific epithet ‘*peruviensis’* is derived from the country where the samples were collected.

#### Ecology and distribution

The species is known from the area around the provinces of Utcubamba (5°40'33.1"S 78°20'23.8"W), Rodríguez de Mendoza (6°26'35.4"S 77°28'44.9"W), and Chachapoyas (6°31'33.0"S 77°48'50.2"W). It is found in the wild in premontane wet forests and montane forests at 1200–1800 m elevation. Plants not cultivated.

#### Specimens examined

Peru, Amazonas, Prov. Utcubamba, Dist. Cajaruro, 5°40'33.1"S, 78°20'23.8"W, 1571 m a.s.l., 19 Oct. 2018, *D*. *Tineo IV19* (CHAX245); Peru, Amazonas, Prov. Rodríguez de Mendoza, Dist. Santa Rosa, 6°26'35.4"S, 77°28'44.9"W, 1887 m a.s.l., 15 Sep. 2018, *D*. *Tineo IV24* (CHAX248); Peru, Amazonas, Prov. Prov. Chachapoyas, Dist. La Jalca, 6°31'33.0"S, 77°48'50.2"W, 2557 m a.s.l., 01 Sep. 2018, *D*. *Tineo IV22* (CHAX246).

#### Remarks

Although *Vasconcellea peruviensis* is morphologically similar to *V*. *monoica*, this species is distinguished from other species of *Vasconcellea* by being monoecious trees. *V*. *peruviensis* is morphologically different from *V*. *monoica* by having globose berries with an acute apex (S4 Table). Additionally, *V*. *peruviensis* grows in sympatry with *V*. *pentalobis* and *V*. *stipulata*. Phylogenetically, *V*. *peruviensis* is also a closely related species to *V*. *monoica*. However, these two species are genetically different at the ITS (0.4%), *matK* (0.3–0.4%), *psb*A-*trn*H (4.6–5%), *rpl*20-*rps*12 (0.3–0.4%), and *trn*L-*trn*F (0.6–0.8%) loci.

Morphologycally, the species of *Vaconcellea* reported from Peru can be distinguished by the following taxonomic key:

1a. Monoecious plants………………………………………………………………..….**2**
1b. Dioecious plants ………………………………………………………………..……**3**
    2a. Petiole length 10–25 cm, seed surface having acute projections …… ..***V*. *monoica***
    2b. Petiole length 25–35 cm, seed surface having large and conical protuberances …………………………..………………………..…………….………***V*. *peruviensis***
3a. Deciduous plants ……………………………………………………………………..**4**
3b. Evergreen plants……………………………………………………………………..**7**
    4a. Color of female flowers from pink to reddish ………………………***V*. *parviflora***
    4b. Color of female flowers green, white, or yellow..……………….……………….**5**
5a. Petiole length 50–60 cm ………………………………..………………***V*. *carvalhoae***
5b. Petiole length 2–10 cm..…………………………………………………………… **6**
    6a. Fruit length 2–8 cm, superior stamens filaments densely pubescent ………………………………………………………………………….***V*. *quercifolia***
    6b. Fruit length 10–18 cm, superior stamens filaments glabrous or slightly pubescent…………………………………………………………………..……….***V*. *candicans***
7a. Seed ellipsoidal-shaped and having pentalobed central leaflet…………***V*. *pentalobis***
7b. Seed fusiform-shaped and lacking pentalobed central leaflet …………………..….**8**
    8a. Stigma having entire apex ………..………………………………………..……**9**
    8b. Stigma having divided apex and fusiform seeds …………..…………………..**11**
9a. Seed surface having longitudinal ridges ………………………………..***V*. *microcarpa***
9b. Seed surface lacking longitudinal ridges ……………………………………….…**10**
    10a. Plant completely covered by minute hairs and having ovoid-prolate fruits …………………………….………………………………………..…..***V*. *pubescens***
    10b. Plants spindle-shaped and strongly angled fruits (fusiform) …… ..***V*. *glandulosa***
11a. Seeds having smooth surfaces with rounded projections……….…..***V*. *weberbaueri***
11b. Seeds having conical protuberances ………………..……………………………..**12**
    12a. Plants having rough-textured leaves and yellow-orange to pink fruits ……………………………….………………………………………….…***V*. *badilloi***
    12b. Plants having glabrous leaves, inflorescence leaflets at its base, and ovoid fruits with acuminate apex ………………………………………..***V*. *chachapoyensis***

## Discussion

The assignment of accurate names for species is crucial, especially for those with confirmed agronomic potential as highland papayas. The taxonomy of these species, which are members of the genus *Vasconcellea*, has been mostly based on morphological characters and multilocus phylogeny, including detailed species descriptions and precise distribution maps [4, 5, 7, 45]. However, the use of additional methodologies and data sets is recommended to establish well-supported boundaries among species [17, 21]. Accordingly, six molecular markers have been used to delimit species in the genus *Vasconcellea* using phylogeny and four DNA-based methods. Although incongruence among some of these methods was observed in our analyses, genetic distance (ABGD, SPN), a coalescence method (BPP), and the multilocus phylogeny supported 22*–*25 different species in *Vasconcellea*, including five new species from northern Peru.

### Integrative approach

Our six loci phylogeny resulted in topology incongruence mainly to single loci phylogenies. However, multilocus sequence data are pivotal for the establishment of robust species delimitations [46, 47]. Incomplete lineage sorting, horizontal gene transfer, gene duplication and loss, hybridization, or recombination are probable explanations for this discordance [48]. Our data provided molecular evidence of hybridization, but natural processes such as introgression, chloroplast capture, selection, differentiation, mutations, and human selection might have all played a part generating evolving hybridizing species complexes in *Vasconcellea* [14, 20, 48, 49]. According to our multilocus phylogeny, 24 species (including 5 new species) were molecularly confirmed in *Vasconcellea* and are distributed in two main lineages, although previous studies grouped them into 3 clades [9, 44, 50]. One of these lineages, labelled clade 1 by d’Eeckenbrugge et al. [44], is composed of six taxa, including three new species, *V*. *badilloi*, *V*. *chachapoyensis*, and *V*. *pentalobis*. The restricted distribution of this lineage confirms its endemism to southern Ecuador and northern Peru with a high level of introgression history and sympatry [44]. The other lineage is composed of 18 species, including two new species, *V*. *carvalhoae* and *V*. *peruviensis*. This lineage contained clades 2 and 3 of d’Eeckenbrugge et al. [44], which are composed of specimens from different taxa but belong to sympatric populations with high morphological diversity related to hybrid segregation and phenotypic plasticity [14]. Strikingly, these two evolutionary lineages in *Vasconcellea* are molecularly well distinguished clades that can be considered two different genera. However, the lack of additional diagnostic features suggests that further analyses (e.g., anatomical observations on the basis of ultrastructure of vegetative and reproductive tissues and chemotaxonomic evaluations) must be accomplished before recognizing them as separate genera.

Regarding the genetic distance methods, similar results to those from the multilocus phylogeny were obtained by ABGD and SPN when delimiting *Vasconcellea* species. The additional putative species identified with these methods mainly resulted from the split of *V*. *chachapoyensis*, *V*. *pentalobis*, and *V*. *peruviensis* with the markers ITS, *psb*A-*trn*H, and *trn*L-*trn*F. This might suggest that these species encompass cryptic lineages [17] as a consequence of the initial hybridization process, but these splits are not supported by the multilocus phylogeny and BPP analyses dismissing crypticism.

In the coalescent methods, the presence of gene flow due to the high hybridization levels in different species of *Vasconcellea* had negative impacts, particularly on the GMYC model [51]. The GMYC model usually produces false positives and complex false positives when delimiting different taxa that have low or high magnitudes of gene flow, respectively [52, 53]. Nevertheless, the validation of BPP supports the status of the species recognized by the multilocus phylogeny (posterior probabilities, pp 0.61–0.99, S3 Table) and did not support those split or merged taxa by GMYC (pp lower than 0.29, S3 Table). Moreover, the additional species delimited by BPP is an unidentified *Vasconcellea* from Peru (IV17, IV18, IV20, IV21) which lacks of support to be considered a different entity. Therefore, sistership between this species and *V*. *stipulata* is not confirmed, suggesting that they might be conspecific. Additional specimens of this unidentified taxon should be sequenced to confirm its taxonomical status. The performance in empirical studies of the genetic and coalescent methods tends to undersplit and oversplit species, respectively [53–56]. However, our results suggest that ABGD and BPP are appropriate for determining diversity in *Vasconcellea* by recognizing those well-supported clades delimited by the multilocus phylogeny.

In our study, *V*. *pentalobis* and *V*. *peruviensis* were morphologically distinguished by their pentalobed central lobes and monoic inflorescence, respectively. Traits related to leaf (petiole length), female flowers (color, stigma shape), seeds (shape and texture), and fruits (length) slightly differentiated the other three new species. For instance, *V*. *badilloi* and *V*. *chachapoyensis* have divided stigmas and conical protuberances on seed surfaces, while *V*. *carvalhoae* has an entire stigma and rounded projections on seed surfaces. The absence of robust morphological distinction traditionally occurs in organisms that lack complex structures such as fungi or algae, but the high phenotypic plasticity and hybridization scenarios in *Vasconcellea* might explain this absence in these plants [9, 44]. This morphological indistinctiveness among some *Vasconcellea* species was overcome by the application of molecular methods in plant taxonomy. In addition, our multilocus data, ABGD, and BPP analyses suggested conspecificity between *V*. *goudotiana*/*V*. *sphaerocarpa* as well as *V*. *pubescens*/*V*. *sprucei*. Therefore, *V*. *sphaerocarpa* (García-Barr. & Hern-Cam, 1958 in Badillo [7]), and *V*. *sprucei* (Badillo [57]) might be synonymized with *V*. *goudotiana* (Triana & Planch, 1873 in Badillo [7]) and *V*. *pubescens* (de Candolle [58]), respectively, on the basis of the principle of priority. However, further studies should delimit the relationships of those taxa including analyses of new material collected from type localities. Although several chloroplast and nuclear sequences have been used for assessing inter- and intraspecific relationships among species of Caricaceae [2, 9, 14, 20], only ITS and *trn*L-*trn*F intergenic showed better resolution for distinguishing species based on phylogeny and species delimitation methods. This suggests that initial screening regarding the diversity of *Vasconcellea* should include amplification of these markers. The segregation of five new species confirmed that phylogenetic diversity and DNA-species delimitation methods could be used to discover taxa within traditionally defined species [15, 17, 59].

## Conclusions

The use of an integrative approach to analyse diversity, including DNA-based delimitation methods, allowed the establishment of boundaries among species with morphological diversity, such as *Vasconcellea*, and thus provided support for the description of new taxa or validated the taxonomic uncertainty of other *Vasconcellea* members. Our results demonstrated that the congruence among different methodologies applied in this integrative study (i.e., morphology, multilocus phylogeny, genetic distance, coalescence methods) are more likely to prove reliably supported species boundaries. Therefore, ABGD, BPP, and multilocus phylogeny are pivotal when establishing species boundaries in *Vasconcellea*.

## Supporting information

S1 Fig Phylogenetic tree based on maximum likelihood inference of combined *matK*, *psbA*-*trnH*, *rbcL*, *trnL*-*trnF* data. Combination of markers were selected on the basis of high genetic pairwise divergence. Value above branches = Maximum likelihood bootstrap values (BS). The scale bar indicates the number of nucleotide substitution per site.
(JPG)

S2 Fig Phylogenetic tree based on maximum likelihood inference of combined *psbA*-*trnH*, *rbcL*, *trnL*-*trnF* data. Combination of markers were selected on the basis of high genetic pairwise divergence. Value above branches = maximum likelihood bootstrap values (BS). The scale bar indicates the number of nucleotide substitutions per site.
(JPG)

S3 Fig Phylogenetic tree based on maximum likelihood inference of combined *matK*, *trnL*-*trnF* data. Combination of markers were selected on the basis of high genetic pairwise divergence. Value above branches = maximum likelihood bootstrap values (BS). The scale bar indicates the number of nucleotide substitutions per site.
(JPG)

S4 Fig Phylogenetic tree based on maximum likelihood inference of combined *trnL*-*trnF* data. Marker was selected on the basis of high genetic pairwise divergence. Value above branches = maximum likelihood bootstrap values (BS). Scale bar indicates the number of nucleotide substitutions per site.
(JPG)

S5 Fig Bayesian inference ultrametric gene tree obtained using a prior Yule tree in BEAST with the statistical species delimitation results from GMYC based on ITS (A), *matK* (B) and *psbA*-*trnH* (C).
(JPG)

S6 Fig Bayesian inference ultrametric gene tree obtained using a prior Yule tree in BEAST with the statistical species delimitation results from GMYC based on *rbcL* (A), *rpl20-rps12* (B) and *trnL-trnF* (C).
(JPG)

S7 Fig (JPG)

S1 Table List of primers used in the molecular analyses. (DOCX)

S2 Table Results of the Generalized Mixed Yule-Coalescent (GMYC) analyses under the single threshold model. (DOCX)

S3 Table Highest posterior probabilities of the six-gene Bayesian species delimitation analysis (BPP) by jointing species delimitation and species tree inference. (DOCX)

S4 Table Morphological comparisons among species of the genus *Vasconcellea*. (XLSX)
