# Supplementary figures and images for: An integrative approach reveals five new species of highland papayas (Caricaceae, Vasconcellea) from northern Peru
Source: PLoS One. 2020 Dec 10;15(12):e0242469. doi: 10.1371/journal.pone.0242469 (PMC7728213; doi:10.1371/journal.pone.0242469)

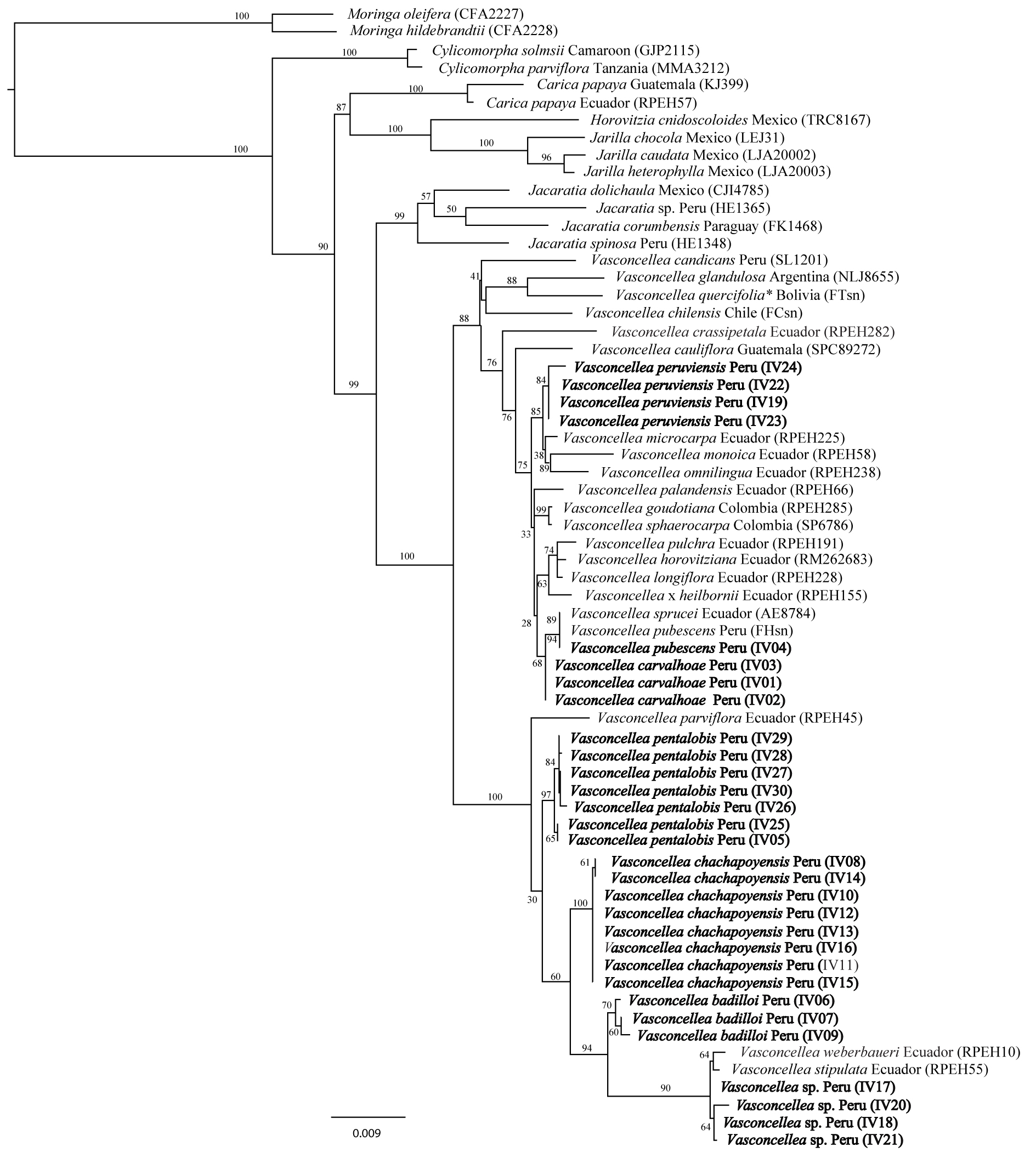

Supplement: S1 Fig — Combination of markers were selected on the basis of high genetic pairwise divergence. Value above branches = Maximum likelihood bootstrap values (BS). The scale bar indicates the number of nucleotide substitution per site. (JPG) [file pone.0242469.s001.jpg]

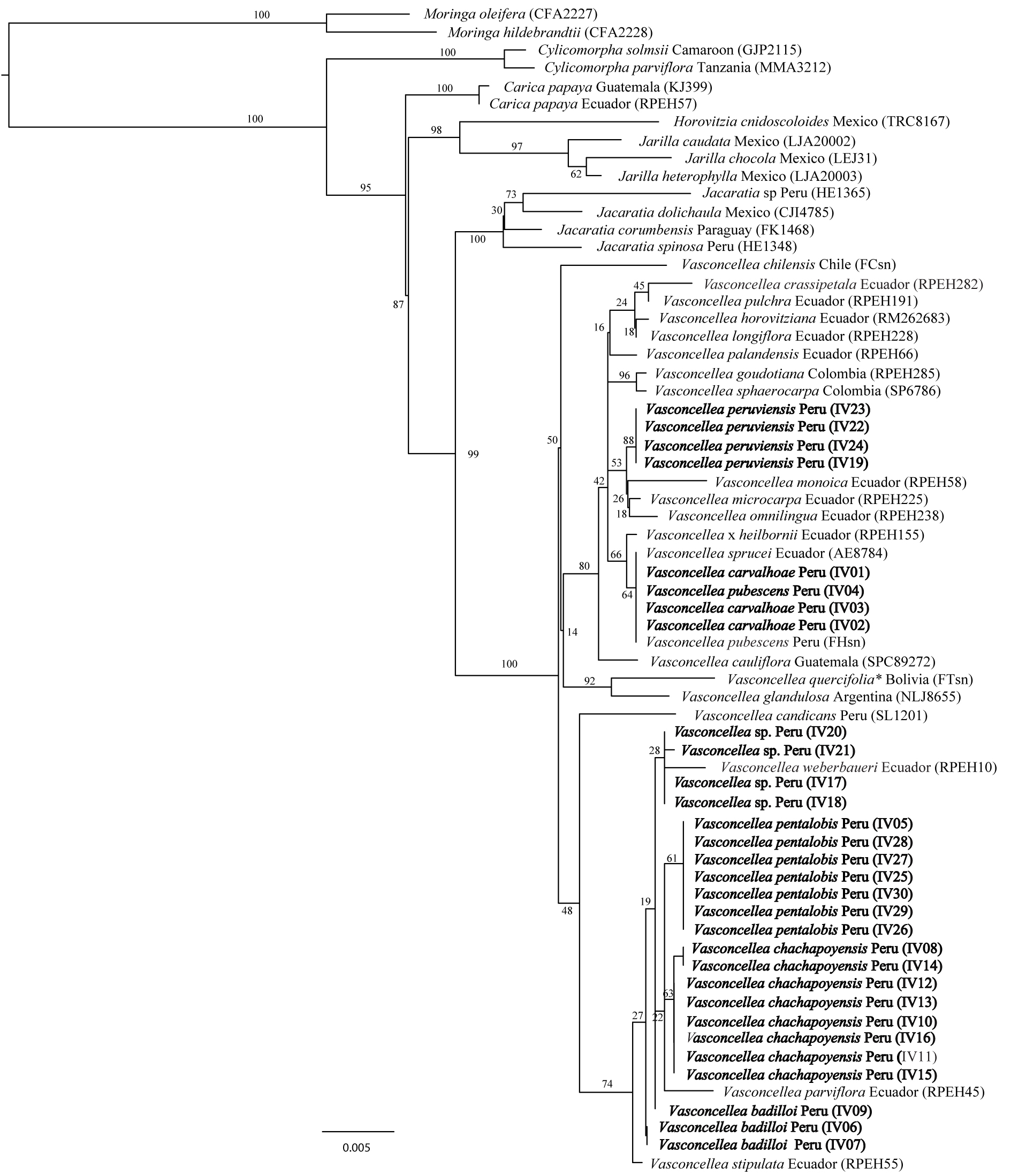

Supplement: S2 Fig — Combination of markers were selected on the basis of high genetic pairwise divergence. Value above branches = maximum likelihood bootstrap values (BS). The scale bar indicates the number of nucleotide substitutions per site. (JPG) [file pone.0242469.s002.jpg]

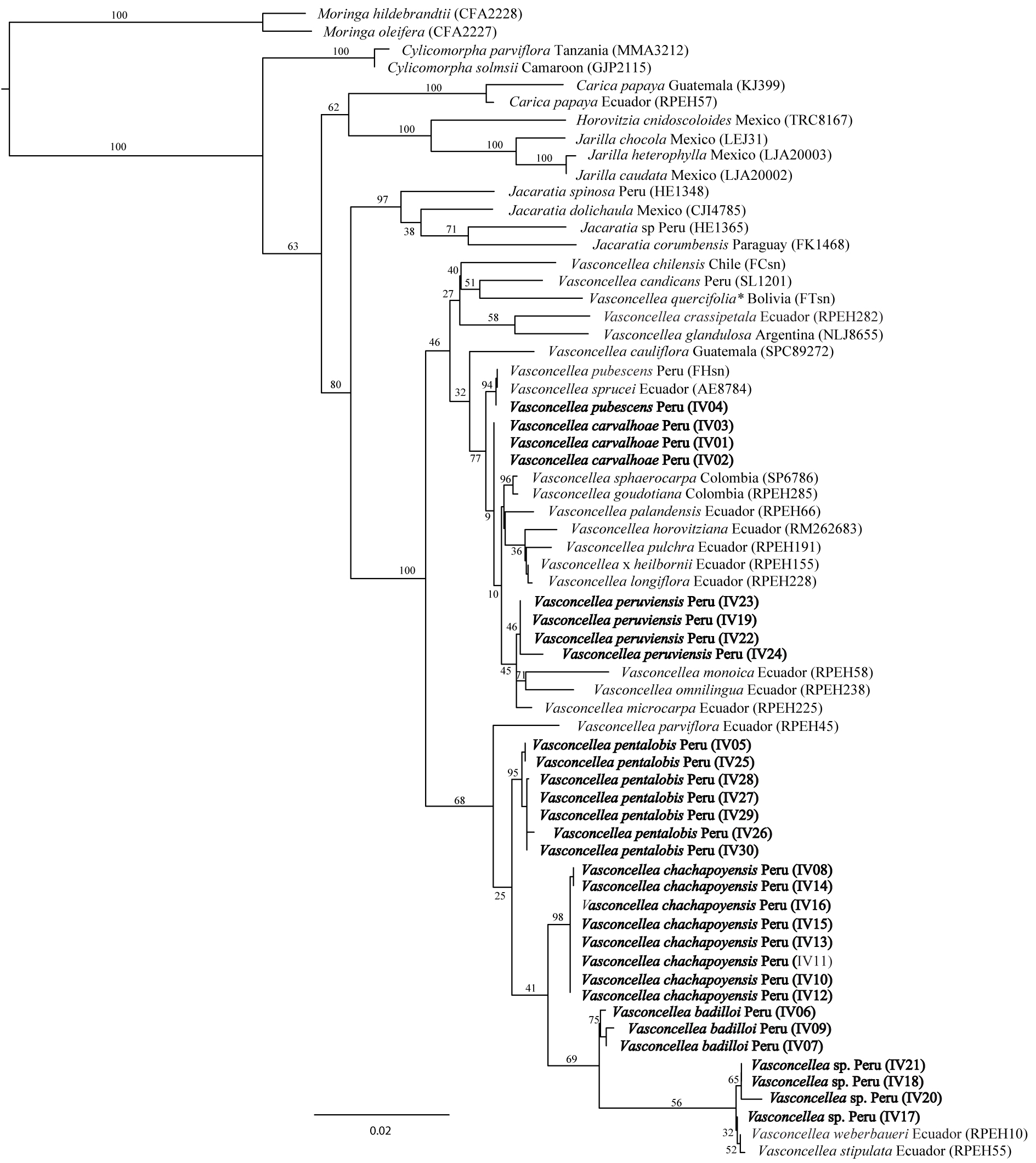

Supplement: S3 Fig — Combination of markers were selected on the basis of high genetic pairwise divergence. Value above branches = maximum likelihood bootstrap values (BS). The scale bar indicates the number of nucleotide substitutions per site. (JPG) [file pone.0242469.s003.jpg]

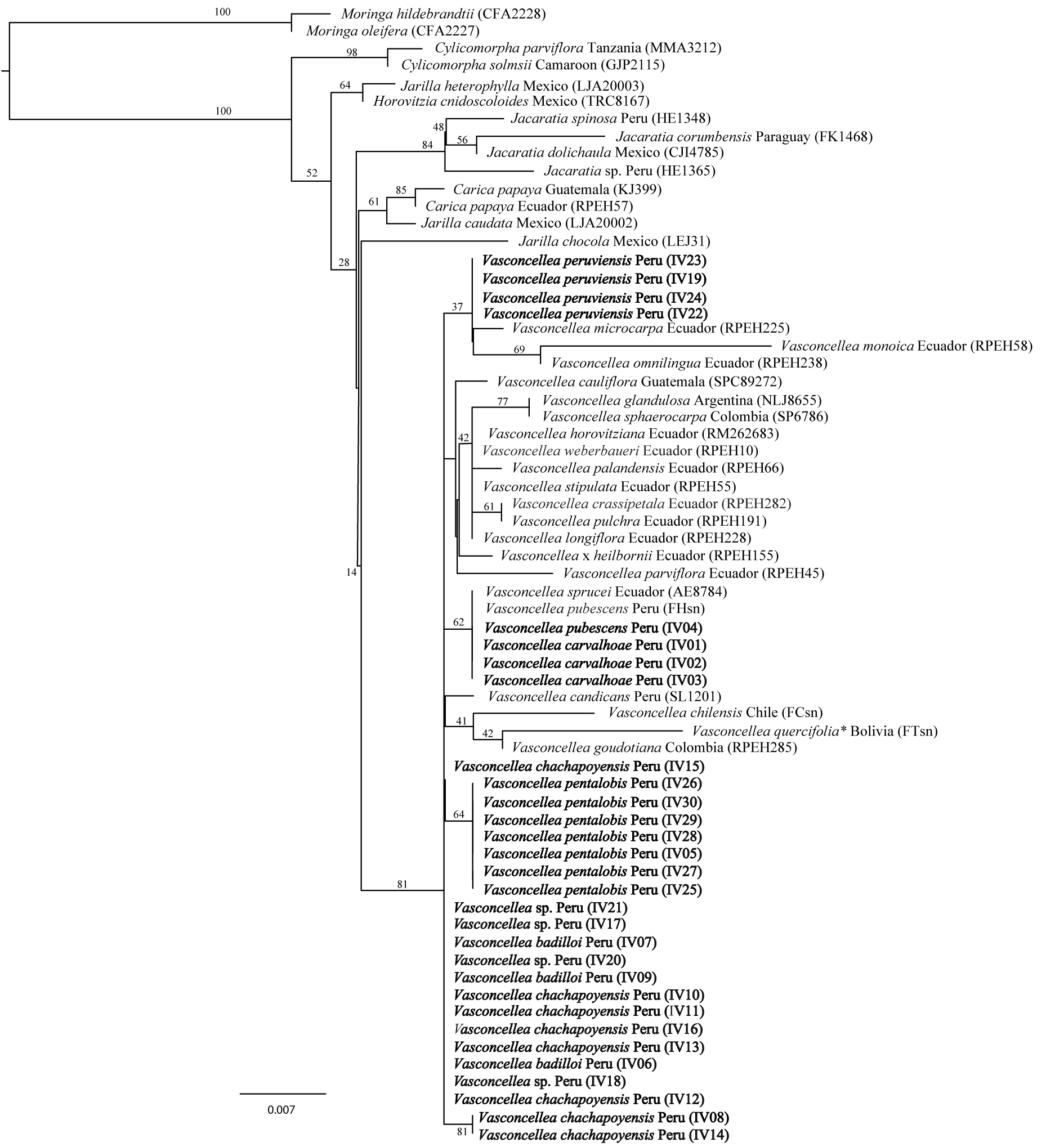

Supplement: S4 Fig — Marker was selected on the basis of high genetic pairwise divergence. Value above branches = maximum likelihood bootstrap values (BS). Scale bar indicates the number of nucleotide substitutions per site. (JPG) [file pone.0242469.s004.jpg]

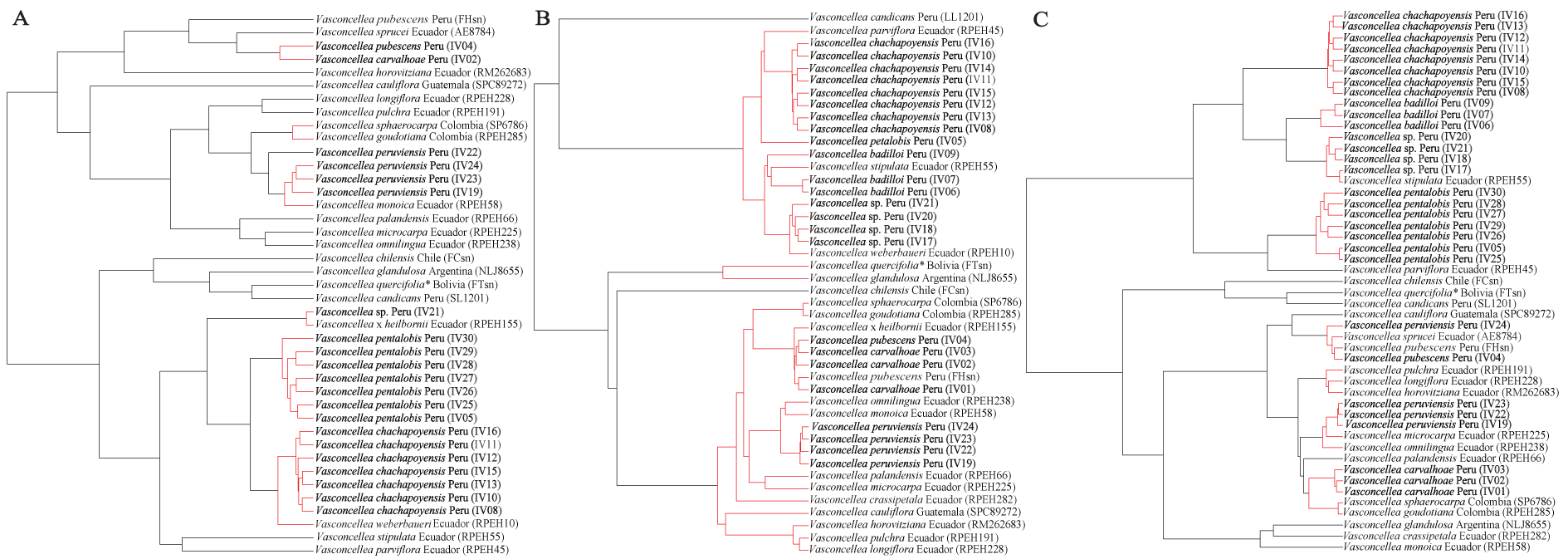

Supplement: S5 Fig — Bayesian inference ultrametric gene tree obtained using a prior Yule tree in BEAST with the statistical species delimitation results from GMYC based on ITS (A), matK (B) and psbA-trnH (C). (JPG) [file pone.0242469.s005.jpg]

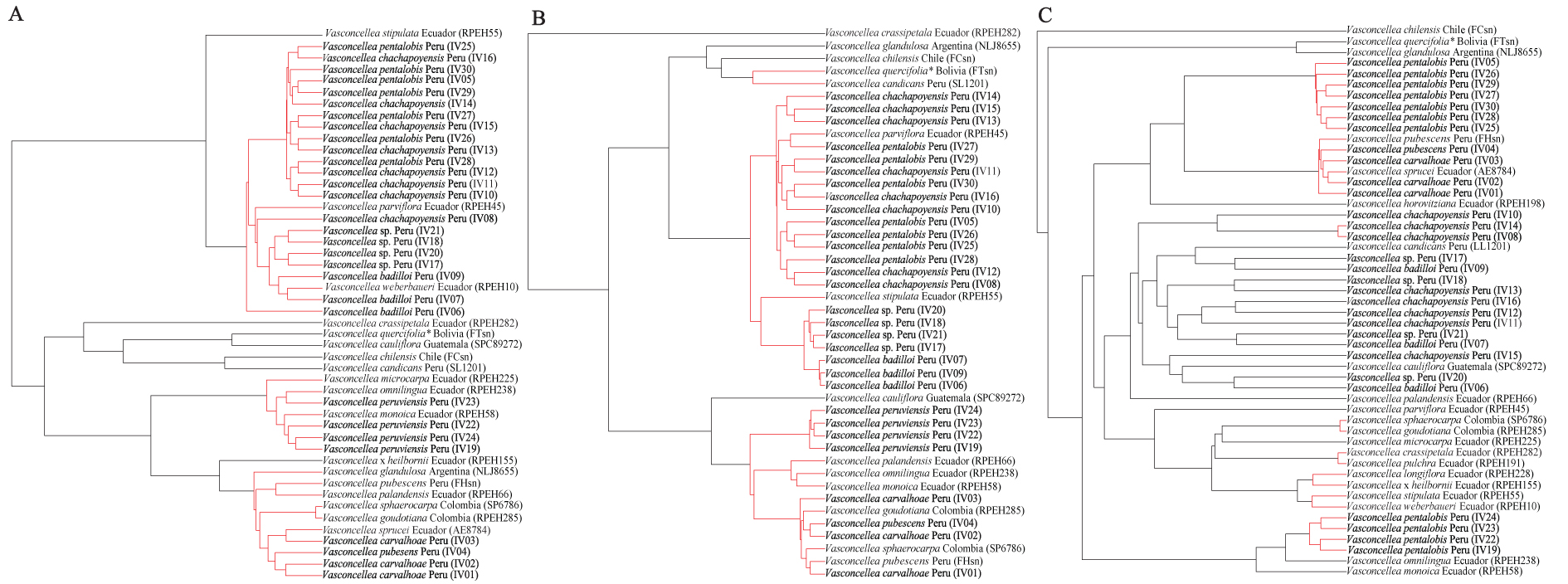

Supplement: S6 Fig — Bayesian inference ultrametric gene tree obtained using a prior Yule tree in BEAST with the statistical species delimitation results from GMYC based on rbcL (A), rpl20-rps12 (B) and trnL-trnF (C). (JPG) [file pone.0242469.s006.jpg]
