# Supplementary material for: An integrative approach reveals five new species of highland papayas (Caricaceae, Vasconcellea) from northern Peru
Source: PLoS One. 2020 Dec 10;15(12):e0242469. doi: 10.1371/journal.pone.0242469 (PMC7728213; doi:10.1371/journal.pone.0242469)
Supplement: S1 Table — (DOCX) [file pone.0242469.s008.docx]

**S1 Table.** List of primers used in the molecular analyses.

| **Marker** | **Size (bp)** | **Primers sequences** | **References** |
| --- | --- | --- | --- |
| ITS | 700 | F: 5'-TCCGTAGGTGAACCTGCGG-3' | White et al. (1990) |
|  |  | R: 5'- TCCTCCGCTTATTGATATGC-3' |  |
| *mat*K | 1500 | F: 5'-CTATATCCACTTATCTTTCAGGAGT-3' | Ooi et al. (1996) |
|  |  | R: 5'-AAAGTTCTAGCACAAGAAAGTCGA-3' |  |
| *psb*A-*trn*H | 450 | F: 5'-GTTATGCATGAACGTAATGCTC-3' | Sang et al. (1997) |
|  |  | R: 5'- CGCGCATGGTGGATTCACAAATC-3' |  |
| *rbc*L | 1400 | F: 5'-ATGTCACCACAAACAGAAACTAAAGC-3' | Chase et al. (1993) |
|  |  | R: 5'- CTTTTAGTAAAAGATTGGGCCGAG-3' |  |
| *rpl*20-*rps*12 | 800 | F: 5'-TTTGTTCTACGTCTTCGAGC-3' | Hamilton (1999) |
|  |  | R: 5'- GTCGAGGAACATGTACTAGG-3' |  |
| *trn*L-*trn*F | 550 | F: 5'-GGTTCAAGTCCCTCTATCCC-3' | Taberlet et al. (1991) |
|  |  | R: 5'- ATTTGAACTGGTGACACGAG-3' |  |
