# Supplementary material for: An integrative approach reveals five new species of highland papayas (Caricaceae, Vasconcellea) from northern Peru
Source: PLoS One. 2020 Dec 10;15(12):e0242469. doi: 10.1371/journal.pone.0242469 (PMC7728213; doi:10.1371/journal.pone.0242469)
Supplement: S2 Table — (DOCX) [file pone.0242469.s009.docx]

**S2 Table.** Results of the Generalized Mixed Yule-Coalescent (GMYC) analyses under the single threshold model.

| **Gene** | ***N*_CLUSTER_ (CI)** | ***N*_GMYC_ (CI)** | ***L*_NULL_** | ***L*_GMYC_** | **Λ** | ***P*** | **Threshold** |
| --- | --- | --- | --- | --- | --- | --- | --- |
| ITS | 6 (1-9) | 22 (1-40) | 243.0432 | 243.3126 | 0.538894 | 0.7638019n.s. | -0.006172397 |
| *mat*K | 3 (2-8) | 5 (2-20) | 358.5711 | 360.8440 | 4.545724 | 0.1030169n.s. | -0.002974298 |
| *psb*A-*trn*H | 8 (6-11) | 17 (14-22) | 298.8308 | 301.8699 | 6.078120 | 0.04787987* | -0.01156442 |
| *rbc*L | 3 (3-5) | 10 (6-12) | 437.1164 | 443.6223 | 13.01184 | 0.001494568** | -0.000691222 |
| *rpl*20*-rps*12 | 3 (1-10) | 7 (1-21) | 363.5084 | 365.2877 | 3.558698 | 0.168748n.s. | -0.001912292 |
| *trn*L*-trn*F | 8 (1-9) | 32 (2-33) | 343.7470 | 346.0413 | 4.588700 | 0.1008269n.s. | -0.002613999 |

Abbreviation: N_CLUSTER_, number of GMYC lineages with more than one specimens; N_GMYC_, number of GMYC lineages; CI, confidence interval; L_NULL_, likelihood of the null model; L_GMYC_, likelihood of the GMYC model; Λ, likelihood ratio; P, P values of the likelihood ratio test; Threshold, the threshold line between speciation and coalescent process.
