# Supplementary material for: An integrative approach reveals five new species of highland papayas (Caricaceae, Vasconcellea) from northern Peru
Source: PLoS One. 2020 Dec 10;15(12):e0242469. doi: 10.1371/journal.pone.0242469 (PMC7728213; doi:10.1371/journal.pone.0242469)
Supplement: S3 Table — (DOCX) [file pone.0242469.s010.docx]

**S3 Table.** Highest posterior probabilities of the six-gene Bayesian species delimitation analysis (BPP) by jointing species delimitation and species tree inference.

| **Species name** | **Run1** | **Run2** | **Run3** | **Run4** | **Run5** | **Average** |
| --- | --- | --- | --- | --- | --- | --- |
| ***V. badilloi*** | 0.638 | 0.627 | 0.601 | 0.612 | 0.579 | 0.611 |
| *V. candicans* | 0.991 | 0.993 | 0.994 | 0.997 | 0.995 | 0.994 |
| ***V. carvalhoae*** | 0.802 | 0.772 | 0.857 | 0.738 | 0.674 | 0.769 |
| *V. cauliflora* | 0.991 | 0.987 | 0.992 | 0.992 | 0.988 | 0.990 |
| ***V. chachapoyensis*** | 0.562 | 0.687 | 0.689 | 0.665 | 0.680 | 0.657 |
| *V. chilensis* | 0.996 | 0.994 | 0.994 | 0.994 | 0.997 | 0.995 |
| *V. crassipetala* | 0.767 | 0.735 | 0.790 | 0.828 | 0.744 | 0.773 |
| *V. glandulosa* | 0.988 | 0.992 | 0.990 | 0.994 | 0.991 | 0.991 |
| *V. goudotiana/sphaerocarpa* | 0.666 | 0.664 | 0.649 | 0.673 | 0.681 | 0.667 |
| *V. x heilbornii* | 0.805 | 0.784 | 0.947 | 0.868 | 0.891 | 0.859 |
| *V. horovitziana* | 0.681 | 0.646 | 0.665 | 0.692 | 0.651 | 0.667 |
| *V. longiflora* | 0.609 | 0.615 | 0.592 | 0.627 | 0.581 | 0.605 |
| *V. microcarpa* | 0.943 | 0.936 | 0.917 | 0.953 | 0.933 | 0.936 |
| *V. monoica* | 0.967 | 0.966 | 0.974 | 0.975 | 0.971 | 0.971 |
| *V. omnilingua* | 0.955 | 0.948 | 0.966 | 0.957 | 0.951 | 0.955 |
| *V. palandensis* | 0.988 | 0.992 | 0.989 | 0.991 | 0.989 | 0.990 |
| *V. parviflora* | 0.999 | 0.999 | 0.998 | 0.998 | 1.000 | 0.999 |
| ***V. pentalobis*** | 0.587 | 0.681 | 0.704 | 0.683 | 0.702 | 0.671 |
| ***V. peruviensis*** | 0.635 | 0.636 | 0.589 | 0.615 | 0.619 | 0.619 |
| *V. pubescens/ sprucei* | 0.635 | 0.636 | 0.689 | 0.615 | 0.619 | 0.639 |
| *V. pulchra* | 0.596 | 0.590 | 0.603 | 0.643 | 0.573 | 0.601 |
| *V. quercifolia* | 0.988 | 0.988 | 0.988 | 0.991 | 0.990 | 0.989 |
| *V. stipulata* | 0.712 | 0.765 | 0.694 | 0.741 | 0.666 | 0.716 |
| *V. weberbaueri* | 0.699 | 0.721 | 0.749 | 0.759 | 0.729 | 0.731 |
| ***Vasconcellea* sp.** | 0.612 | 0.546 | 0.564 | 0.589 | 0.608 | 0.584 |
| Splitting a species | 0.237 | 0.271 | 0.345 | 0.278 | 0.331 | 0.292 |
| More than 2 species cluster | 0.157 | 0.107 | 0.114 | 0.198 | 0.140 | 0.143 |
