# Supplementary material for: An integrative approach reveals five new species of highland papayas (Caricaceae, Vasconcellea) from northern Peru
Source: PLoS One. 2020 Dec 10;15(12):e0242469. doi: 10.1371/journal.pone.0242469 (PMC7728213; doi:10.1371/journal.pone.0242469)
Supplement: Attachment — Submitted filename: 2 Response to Reviewers.docx [file pone.0242469.s013.docx]

Dear Dr. Himanshu Sharma

Academic Editor

PLOS ONE

PONE-D-20-23252R1

**"An integrative approach reveals five new species of highland papayas (Caricaceae, Vasconcellea) from northern Peru"**

Please, kindly find our response.

**Editor's Comments to Authors**

**Additional Editor Comments:** The manuscript entitled PONE-D-20-23252R1 An integrative approach reveals five new species of highland papayas (Caricaceae, Vasconcellea) from northern Peru has been extensively reviewed by all the reviewers and they agreed for the acceptance, but one reviewer has raised query that they have not provided the relationship of studied species on the basis of morphology in the form of deprogram which can provide additional evidence for the study and also some grammatical errors which needs to be corrected before the final approval.

- **COMMENTAND FIXED:** In the previous review, we have included a table summarizing morphological features and a dichotomous key features among species of *Vasconcellea* as additional evidence for the morphological analyses since the dendrogram is not showing any additional evidence as you can notice in the attached figure (Fig. 1). This analyses poorly resolved several species as only one (e.g. *V. sprucei, V. pulchra, V. candicans, V. glandulosa*) and additionally it resulted in very low bootstrap support (blue numbers). We kindly ask you to reconsider the inclusion of this information in the manuscript.

Fig. 1. Dendrogram based on a hierarchical cluster analysis for 25 species of *Vasconcellea.* This dendrogram was drawn based on features Table S4 and a hierarchical cluster analysis using single linkage (nearest-neighbour) procedure using DARwin computer software version 6 for the 25 species of *Vasconcellea.*

In addition, grammatical errors pointed out by reviewer 2 have been fixed.

**Reviewers' Comments to Authors**

**Reviewer #2:** Although, authors have addressed all the raised issues but still they have not provided the relationship of studied species on the basis of morphology in the form of deprogram which can provide additional evidence for the study. I also advise authors to revise and thoroughly examine the language of manuscript to avoid ambiguity and to make your story more specific and objective oriented.

- **COMMENTAND FIXED:** In the previous review, we have included a table summarizing morphological features and a dichotomous key features among species of *Vasconcellea* as additional evidence for the morphological analyses since the dendrogram is not showing any additional evidence as you can notice in the attached figure (Fig. 1). This analyses poorly resolved several species as only one (e.g. *V. sprucei, V. pulchra, V. candicans, V. glandulosa*) and additionally it resulted in very low bootstrap support (blue numbers). We kindly ask you to reconsider the inclusion of this information in the manuscript.

Fig. 1. Dendrogram based on a hierarchical cluster analysis for 25 species of *Vasconcellea.* This dendrogram was drawn based on features Table S4 and a hierarchical cluster analysis using single linkage (nearest-neighbour) procedure using DARwin computer software version 6 for the 25 species of *Vasconcellea.*

For example in conclusion : starting of conclusion is not relevant neither it contain concrete information of your work. in line 678 what do you mean by others? please also check entire manuscript for such type of use of words and be specific. After fixing these issues manuscript can be accepted.

- **COMMENTAND FIXED:** Grammatical errors pointed out by reviewer 2 have been fixed. Starting line of conclusion was removed and lines 58, 273, 590, 636, 654, 659, 673, and 678.

Thank you for your consideration,

Sincerely,

Ph.D. Danilo E. Bustamante

Universidad Nacional Toribio Rodríguez de Mendoza

danilo.bustamante@untrm.edu.pe
